# Structural and functional analysis of coral Hypoxia Inducible Factor

**DOI:** 10.1371/journal.pone.0186262

**Published:** 2017-11-08

**Authors:** Didier Zoccola, Jonas Morain, Gilles Pagès, Natacha Caminiti-Segonds, Sandy Giuliano, Sylvie Tambutté, Denis Allemand

**Affiliations:** 1 Centre Scientifique de Monaco, Department of Marine Biology, 8 Quai Antoine Ier, Principality of Monaco; 2 Centre Scientifique de Monaco, Department of Medical Biology, 8 Quai Antoine Ier, Principality of Monaco; 3 University Cote d’Azur, Institute for research on cancer and aging of Nice (IRCAN), CNRS UMR 7284/INSERM U 1081, Nice, France; 4 Centre Scientifique de Monaco, 8 Quai Antoine Ier, Principality of Monaco; University of Hawaii System, UNITED STATES

## Abstract

Tissues of symbiotic Cnidarians are exposed to wide, rapid and daily variations of oxygen concentration. Indeed, during daytime, intracellular O_2_ concentration increases due to symbiont photosynthesis, while during night, respiration of both host cells and symbionts leads to intra-tissue hypoxia. The Hypoxia Inducible Factor 1 (HIF-1) is a heterodimeric transcription factor used for maintenance of oxygen homeostasis and adaptation to hypoxia. Here, we carried out a mechanistic study of the response to variations of O_2_ concentrations of the coral model *Stylophora pistillata*. *In silico* analysis showed that homologs of HIF-1 α (SpiHIF-1α) and HIF-1β (SpiHIF-1β) exist in coral. A specific SpiHIF-1 DNA binding on mammalian Hypoxia Response Element (HRE) sequences was shown in extracts from coral exposed to dark conditions. Then, we cloned the coral HIF-1α and β genes and determined their expression and transcriptional activity. Although HIF-1α has an incomplete Oxygen-dependent Degradation Domain (ODD) relative to its human homolog, its protein level is increased under hypoxia when tested in mammalian cells. Moreover, co-transfection of SpiHIF-1α and β in mammalian cells stimulated an artificial promoter containing HRE only in hypoxic conditions. This study shows the strong conservation of molecular mechanisms involved in adaptation to O_2_ concentration between Cnidarians and Mammals whose ancestors diverged about 1,200–1,500 million years ago.

## Introduction

Corals (Anthozoa, Scleractinia) play a pivotal role in marine ecosystems and are at the basis of the foundation of coral reefs. These Metazoans live in oligotrophic water and thus in a nutrient-poor environment. To adapt to this environment, corals have acquired, through evolution, photosynthetic symbionts, Dinoflagellates from the *Symbiodinium* genera. The most important benefit acquired by this association is nutritional, since symbionts transfer to the host most of the organic carbon produced by photosynthesis to their host, contributing around 90% of their carbon and energy needs [1]. Due to the presence of intracellular Dinoflagellates, symbiotic Cnidarians are exposed to wide, rapid and daily variations of oxygen concentration. Indeed, during daytime, intracellular O_2_ concentration increases due to the symbiont’s photosynthetic process, while during nighttime, respiration of both host cells and symbionts leads to intra-tissue hypoxia [2]. Corals do not appear to be damaged by the rapid transition between hypoxia and hyperoxia and are well adapted to these huge variations. This suggests that such animals may be useful comparative models to examine the susceptibility and resistance to hyperoxia-hypoxia transition, as well as oxygen homeostasis. Although Cnidarian adaptation to hyperoxia has been the subject of numerous studies (see [3]), knowledge on the mechanisms of adaptation to hypoxia is still lacking.

It is well established that, in higher eukaryotes, maintenance of oxygen homeostasis and adaptation to hypoxia require a Hypoxia Inducible Factor (HIF), which is a heterodimeric transcription factor composed of an α subunit and a β subunit (the aryl hydrocarbon receptor nuclear translocator—ARNT). HIFα and HIFβ both belong to the basic Helix-Loop- Helix–Per-ARNT-Sim (bHLH–PAS) superfamily [4]. Whereas HIFβ is stable, HIFα is sensitive to oxygen concentration ([5] for review.) In mammals, the oxygen-dependent degradation domain (ODD) of HIF-1α is hydroxylated by prolyl hydroxylase domain (PHD) enzymes under normoxia. These proline residues are highly conserved in other mammalian forms of HIF-1α. Once the proline residue is hydroxylated, the HIF-1α is then recognized by the von Hippel Lindau tumor suppressor (VHL) ubiquitin protein ligase and targeted for ligation-mediated proteasomal degradation [6, 7]. During hypoxia, prolyl hydroxylation is blocked due to decreased levels of oxygen, which leads to the stabilization of HIF-1α and its entry into the nucleus via its nuclear translocator signal motif [8]. Once in the nucleus, HIF-1α dimerizes with HIF-1β to form a functional HIF-1 that binds to the A/GCGTG consensus motif in target gene promoter regions, known as hypoxia-responsive elements (HREs). This initiates the expression of HIF-responsive genes via two independent transactivation domains (N-TAD and C-TAD) [9]. Oxygen availability also regulates HIF-1α activity through another hydroxylation event. This hydroxylation site present on asparagine 803 (Asn/N 803) was identified on the C-TAD of HIF-1α. Hydroxylation on N803 by factor inhibiting hydroxylase (FIH) prevents the interaction of HIF-1α with its coactivators leading to inhibition of HIF-1α transcriptional activity in normoxia [10].

Three HIFα genes have been identified in mammals: HIF-1α, HIF-2α, and HIF-3α. HIF-1α and HIF-2α share significant sequence identity whereas HIF-3α shares relatively low sequence identity with them. HIF-1α and HIF-2α both contain ODD/N-TAD and C-TAD [5] but HIF-3α lacks the C-TAD [11].

Unlike mammals, invertebrates only have a single HIFα gene [12]. Furthermore, the C-TAD asparagine hydroxylation and the FIH-like molecule are absent in *Caenorhabditis elegans* or *Drosophila melanogaster*, but present in *Lottia gigantea* or *Nematostella vectensis* [12].

Here, we carried out a mechanistic study of the hypoxic response of the coral model *Stylophora pistillata*. We showed that HIF-1 exists in coral. Like its mammalian homolog, it is stabilized in hypoxia. In addition, it binds to equivalent DNA motifs. Moreover, corallian HIF-1 stimulates the transcription of an artificial promoter containing HRE sequences in mammalian cells, which strongly suggests a conserved function through evolution.

## Materials and methods

### Experimental animals

Experiments were conducted in a laboratory setting using the zooxanthellate scleractinian coral *Stylophora pistillata*. Colonies were cultivated as indicated previously [13]. To determine the effect of varying oxygen levels on HIFα gene expression, nubbins were sampled in two conditions: light (dissolved oxygen level, ~11 mg/L) and dark (dissolved oxygen level, ~6.4 mg/L).

### Data mining and sequence analysis

Sequences homologous to human HIF-1α and HIF-1β amino acid sequences from NCBI (http://www.ncbi.nlm.nih.gov/protein) were identified amongst *Stylophora pistillata* ESTs [14, 15] and genomic [16] sequences using the TblastN algorithm. For sequence analysis, sequences were scanned for matches against the InterPro protein signature databases, using InterProScan tool (http://www.ebi.ac.uk/interpro/search/sequence-search).

### Cell culture

Human embryonic kidney (HEK) 293T cells were purchased from the American Type Culture Collection (ATCC^®^). They were grown in Dulbecco’s modified Eagle’s medium (DMEM) supplemented with 10% FBS, penicillin(50 units/ml), and streptomycin sulfate (50 pg/ml). Hypoxia treatment was conducted in a hypoxic chamber filled with 1% O_2_. Hypoxic conditions were produced by incubation of cells in a sealed Bug-Box anaerobic workstation (Ruskin Technologies, Jouan). The oxygen in this workstation was maintained at 1–2%, with the residual gas mixture being 93–94% N_2_, and 5% carbon dioxide.

### Transient transfection and luciferase assay

Custom-made gene synthesis was performed by Eurofins. Briefly, coral sequences were submitted to GENEius for human codon usage optimization to improve gene expression in HEK cells. A His-tag coding sequence for SpiHIFα and for SpiHIFβ coding sequence was added at the 5’ end of each gene respectively. The synthetic genes were then subcloned in pIRES2-DsRed-Express (Clontech). Both plasmids were verified by DNA Sanger sequencing.

For luciferase assays, HEK 293T cells were used because they possess high transfection efficiency. Briefly, subconfluent cells were co-transfected using Lipofectamine 2000 (Life Technologies) with 0.2 μg of HIF subunits and, 1 μg of the reporter vector (pRE-tk-LUC) containing three copies of the HRE from the erythropoietin gene [17]. The Renilla control plasmid (0.2 μg—Promega) was cotransfected with the test plasmids to control for the transfection efficiency. Luciferase activity was measured 48h post transfection.

### SpiHIFα antibody production

Polyclonal antibodies against SpiHIFα were produced in rabbit by Eurogentec. Two out of ten rabbits initially screened for absence of cross reactivity with *S*. *pistillata* and HEK cell proteins were selected for the Speedy 28-day program (Eurogentec). Then, antibodies were raised against the following peptides; CKRRSRDAARSRRGQQ (amino-acids 14–28) and CDDFPSPTQSENSTAE (amino-acids 505–519). The antibodies were affinity purified by Eurogentec before use. Antibodies were tested on extracts of HEK cells transfected with plasmids coding for SpiHIFα (S1 Fig).

### Western immunoblot analysis

Cells were lysed in TNET buffer (50 mM Tris-HCl, 100 mM NaCl, 5 mM EDTA, and 1% TritonX-100, pH 7.5) containing protease and phosphatase inhibitors (1 g/ml leupeptin, 1 g/ml pepstatin, 2 M PMSF, 2 g/ml aprotinin, 2.5 M sodium pyrophosphate, 1 M sodium fluoride, and 1 M sodium orthovanadate). Protein concentrations were determined with a BCA Protein Assay kit (Pierce). Homogenized samples were mixed with protein loading buffer and 2-mercaptoethanol (final concentration 5%) and boiled for 5 min. Equal amounts of samples were separated by 7.5% SDS-PAGE and subjected to immune blot analysis. The anti-beta-actin antibody was obtained from Thermo Scientific, the anti-histidine-tag (His-tag) from Serotec, and the anti-myc from Sigma Aldrich.

### Elecrophoretic Mobility Shift Assay (EMSA)

Nuclear extracts of coral were prepared with the NE-PER nuclear extraction reagent (Pierce). Binding reactions contained 3 μg of nuclear extract proteins, buffer (10 mM Tris, pH 7.5, 50 mM KCl, 5 mM MgCl_2_, 1 mM dithiothreitol, 0.05% NP-40, and 2.5% glycerol), 1 μg of poly(dI- dC), and 2 nM of biotin-labeled stranded oligonucleotides W18 (5’-biotin-GCCCTACGTGCTGTCTCA-3’) or M18 (5’-biotin-GCCCTAAAAGCTGTCTCA-3’) [18]. Reactions were incubated at 23°C for 20 min. Competition reactions were performed by adding 200-fold excess unlabeled oligonucleotides to the reaction mixture. Reactions were run on a 5% precasted TBE gel (Biorad) at 100 V for 1 h in a 100 mM Tris borate-EDTA buffer. Reactions were transferred to a Hybond N+ membrane. The biotin-labeled DNA was detected with LightShift chemiluminescent EMSA kit (Pierce).

### Statistics

Results were expressed as the mean ± the standard error (SEM). Statistical significance and p values were determined by the two-tailed Student’s t-test on at least three independent experiments. The results were considered statistically significant when p-value was inferior to 0.05.

## Results

### Characterization of Stylophora HIFα and HIFβ genes

We used genome [16] and transcriptome [14, 15] datasets of *Stylophora pistillata* to make KEGG-assigned functional classification [19]. This method generates BLAST comparisons against the KEGG GENES database, to assign KEGG orthology identities. It allowed us to show that all the known genes included in the HIF pathway are present in *S*. *pistillata*. We focused on the HIF subunits themselves and showed that, as for all known invertebrate genomes [20], *S*. *pistillata* contains a single *HIF*α (*spiHIF*α) and a single *HIFβ* (*spiHIFβ*) gene. Sequence analysis indicated that the HIFα and HIFβ cDNA sequences are 5086 bp and 5264 bp in size respectively and that the HIFα and HIFβ genes contains 11 exons and 17 exons respectively (S1 File).

**Fig 1:**
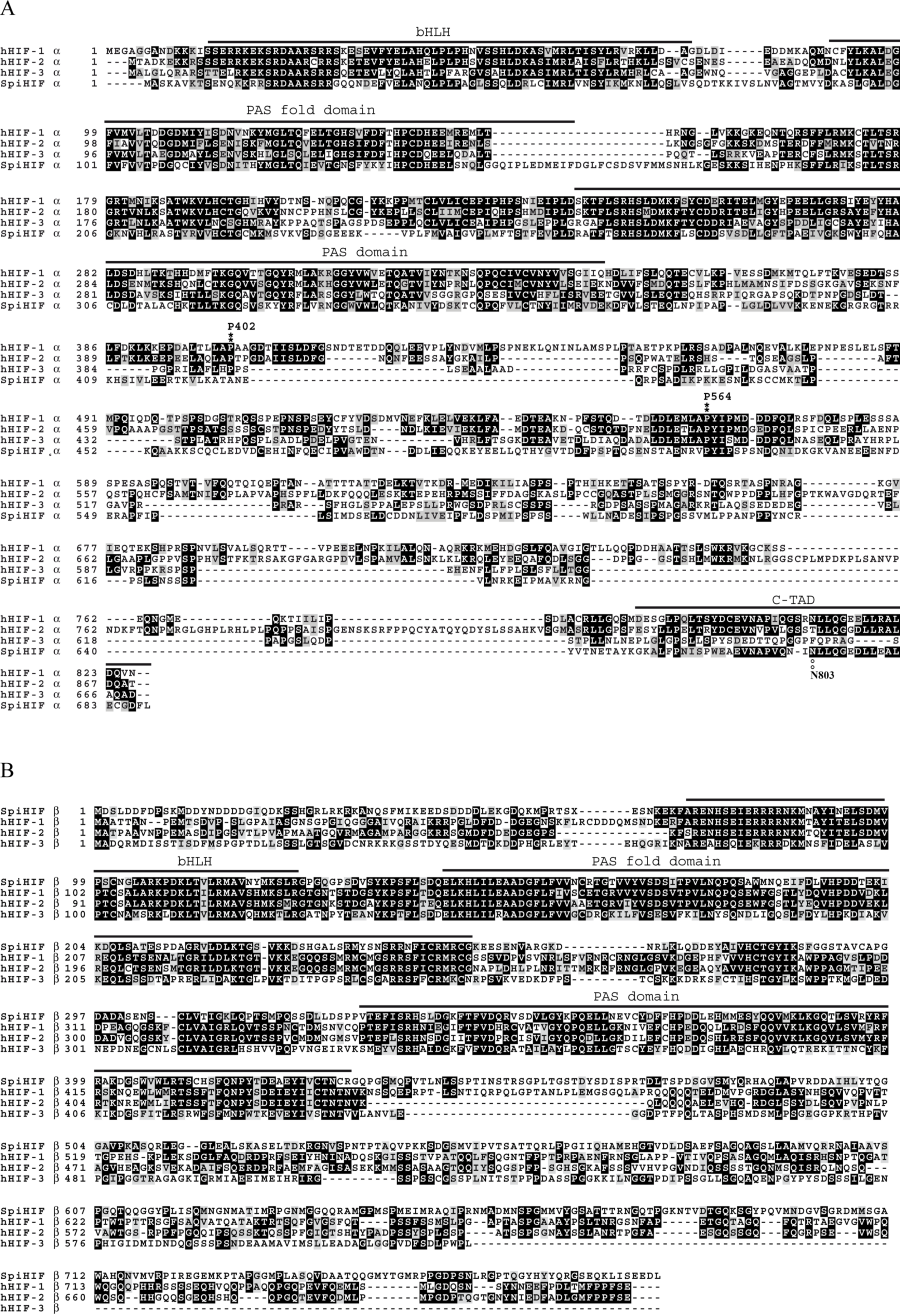
Alignment of human HIF subunit α (A) and β (B) gene products with the deduced amino acid sequence of *Stylophora pistillata* HIF. Black shading shows the sequences that are identical in all four proteins. The different domains are upper lined and annoted. Positions of proline 402 and proline 562 are also noted. The figure was compiled from the NCBI entries: EAW80806 (hHIF-1α), Q99814 (hHIF-2α), Q9Y2N7 (hHIF-3α), P27540 (hHIF-1β), NP_055677 (hHIF-2β), NP_001025443 (hHIF-3β), KY201389 (SpiHIFα and KY201390 (SpiHIFβ).

A ClustalW alignment of SpiHIFα protein with human isoforms HIF-1α, HIF-2α, and HIF-3α is presented in Fig 1A. The best alignment of the deduced amino acid sequence (688 aa in total) was obtained with the human HIF-3α and presented 23% in identity and 39% in conservative substitutions. A conserved domain search on Interpro [21] showed the presence of bHLH (AA 10–74), PAS fold (AA 92–162), PAS domain (AA 263–371) and HIF-1α C-terminal transactivation domains (C-TAD: AA 649–684). Interpro search also showed five putative nuclear import signals (AA 47–67, 104–123, 136–154, 216–235 and 265–282).

Interestingly, in the ODD domain, the proline residue corresponding to P564 in human HIF-1α was present, while the proline residue analogous to P402 was absent in *Stylophora pistillata*. The analogous asparagines residue to N803 in the C-TAD, and hydroxylated by FIH, was detected in SpiHIFα.

Regarding SpiHIFβ, ClustalW alignment with human isoforms HIF-1β, HIF-2β, and HIF-3β is presented in Fig 1B. The best alignment of the deduced amino acid sequence (786 aa in total) was obtained with the human HIF-1β and presented 34% identity and 48% conservative substitutions. A conserved domain search on Interpro [21] showed the presence of bHLH (AA 72–125), PAS fold (AA 145–252), and PAS domains (AA 329–432). Interpro search also showed nine putative nuclear translocator signatures (86–102, 106–126, 136–159, 161–180, 193–211, 239–252, 274–293, 304–320, and 331–348). Because the percentage of similarity between SpiHIFα and its human counterparts was low, and because only one proline hydroxylation site was present in the α subunit, we wanted to assess if HIFα in coral was also regulated by oxygen levels.

### SpiHIFα is regulated by oxygen in HEK cells

**Fig 2:**
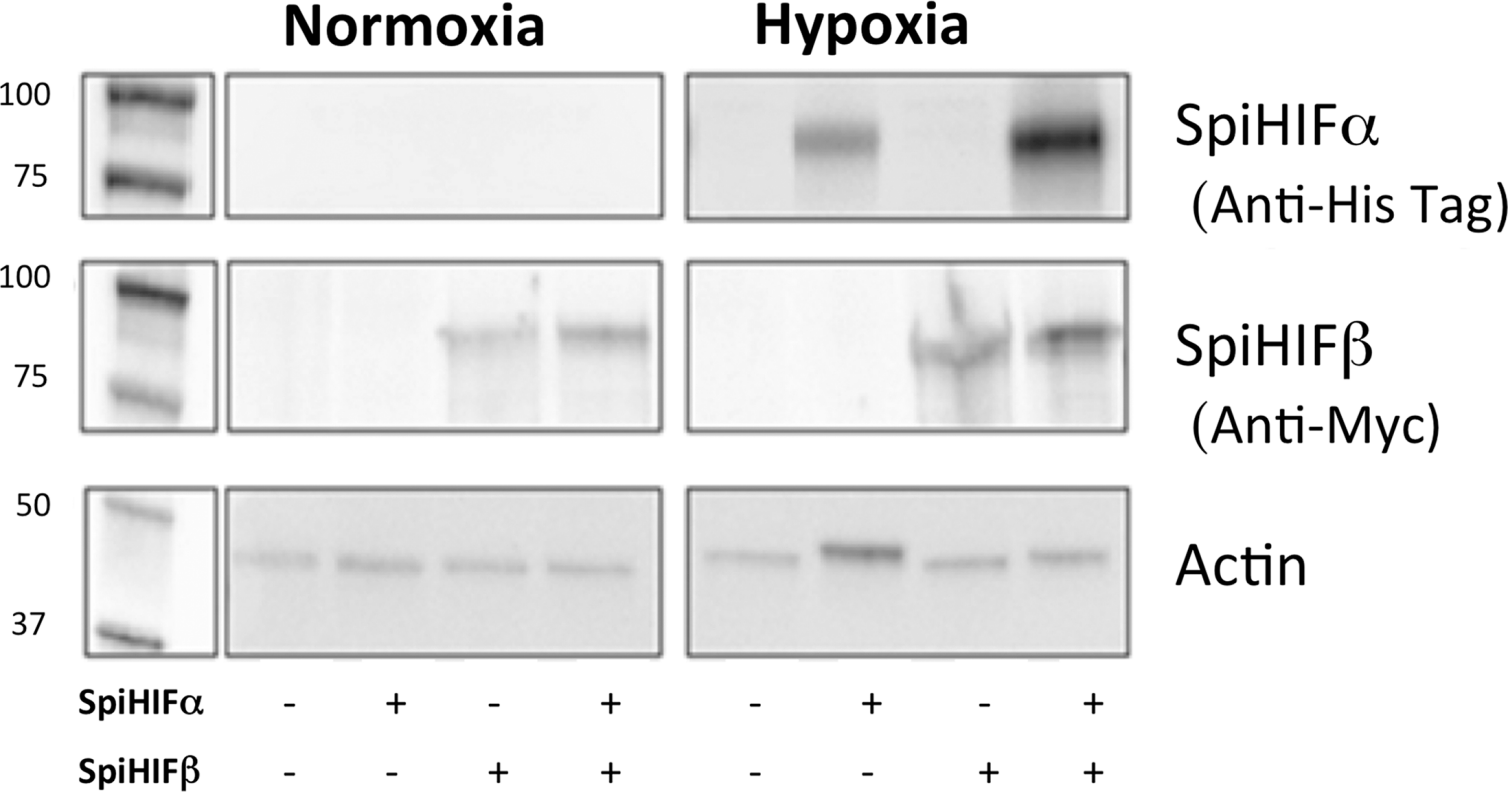
Sp*i*HIFα has a functional ODD domain. HEK293 cells were transfected with 0.1μg of *spiHIF* α or *spiHIFβ* plasmids. 24h post-transfection, cells were maintained in normoxic (21%) or hypoxic (<1%) conditions during 6h. Whole cell extracts migrated within a 7.5% SDS PAGE gel, were analyzed by immuno blot with different antibodies: anti-Histidine tag for SpiHIFα, anti-Myc for SpiHIFβ, and anti-actin as a loading control.

We examined if changes in oxygen levels regulated SpiHIFα at the protein level. For this experiment, we used human HEK 293T cells. The cells were transfected with an empty vector, a His-tagged *spiHIF*α expression plasmid, a Myc-tagged SpiHIFβ expression plasmid or with SpiHIFα and SpiHIFβ expression plasmids (Fig 2). Actin was used as a loading control.

In the His-tagged SpiHIFα-transfected cells, no SpiHIFα was detected in normoxia. In hypoxia, SpiHIFα was detected by anti-His-Tag antibodies (Fig 2). No SpiHIFα was detected in the empty vector–transfected control cells in normoxia or hypoxia. In the Myc-tagged *spiHIFβ*-transfected cells, SpiHIFβ was detected in normoxia and hypoxia. No SpiHIFβ was detected in the empty vector–transfected control cells in normoxia or hypoxia.

These results show that SpiHIFα is regulated by oxygen concentration in HEK cells and probably contains a functional ODD comparable to those of human HIF-1α.

### Hypoxic regulation and binding to HRE of SpiHIFα

**Fig 3:**
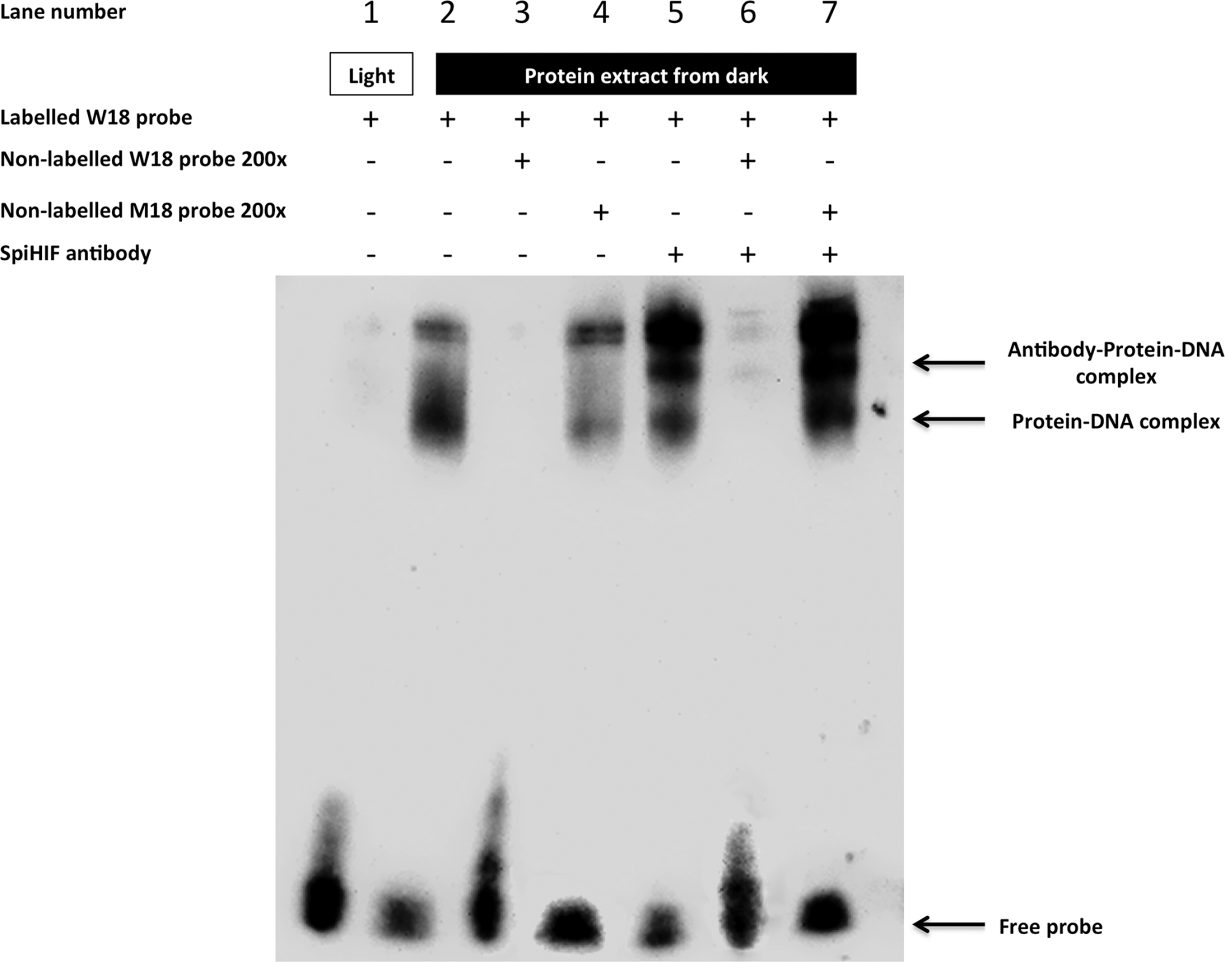
Electrophoretic-Mobility Shift Assay using biotin-labeled W18 probes. Nuclear extracts from *Stylophora pistillata* sampled at light (lane 1) or dark (lane 2 to 7) were incubated with labeled synthetic HRE (W18) then subjected on 5% TBE acrylamide gel. Competition reactions with W18 as competitive probe (lane 3 and 6) or M18 as non-competitive (lane 4 and 7) were performed by adding 200-fold excess unlabeled oligonucleotides. Super shift experiment (lane 5 to 7) was performed mixing anti-SpiHIFα with nuclear extract prior incubation with labelled W18. The complex formed by SpiHIFα and probe (and anti-SpiHIFα when added) is indicated by arrows.

Next, we assessed whether SpiHIFα was sensitive to oxygen levels *in hospite* considering that symbiotic cnidarians are subjected to an abrupt variation of oxygen concentration during day (light) or night (dark) times [22]. Hence, extracts from light or dark exposed corals were used in EMSA experiments using mammalian wild-type HRE (W18) as a probe [18]. Retarded bands were only observed when W18 was incubated with nuclear extracts from coral in dark conditions (Fig 3, lane 2). The binding specificity of the complexes formed were determined by competition with an excess of unlabeled W18 (Fig 3, lane 3) and absence of competition with an excess of an unlabeled probe (M18) containing a 3-bp substitution in the HIF-1 binding site (Fig 3, lane 4). To demonstrate that SpiHIFα is present in the complex, we performed supershift experiments with an antibody directed against SpiHIF. The antibody specificity was tested in Western blot with extracts of SpiHIFα-transfected HEK subjected to hypoxia (S1 Fig). Indeed, anti-SpiHIFα antibodies supershifted part of the complex observed in lane 2 (Fig 3, lane 5). An excess of unlabeled W18 probe prevented the supershift (Fig 3, lane 6), whereas M18 did not (Fig 3, lane 7).

### SpiHIF activates HRE-dependent gene expression

**Fig 4:**
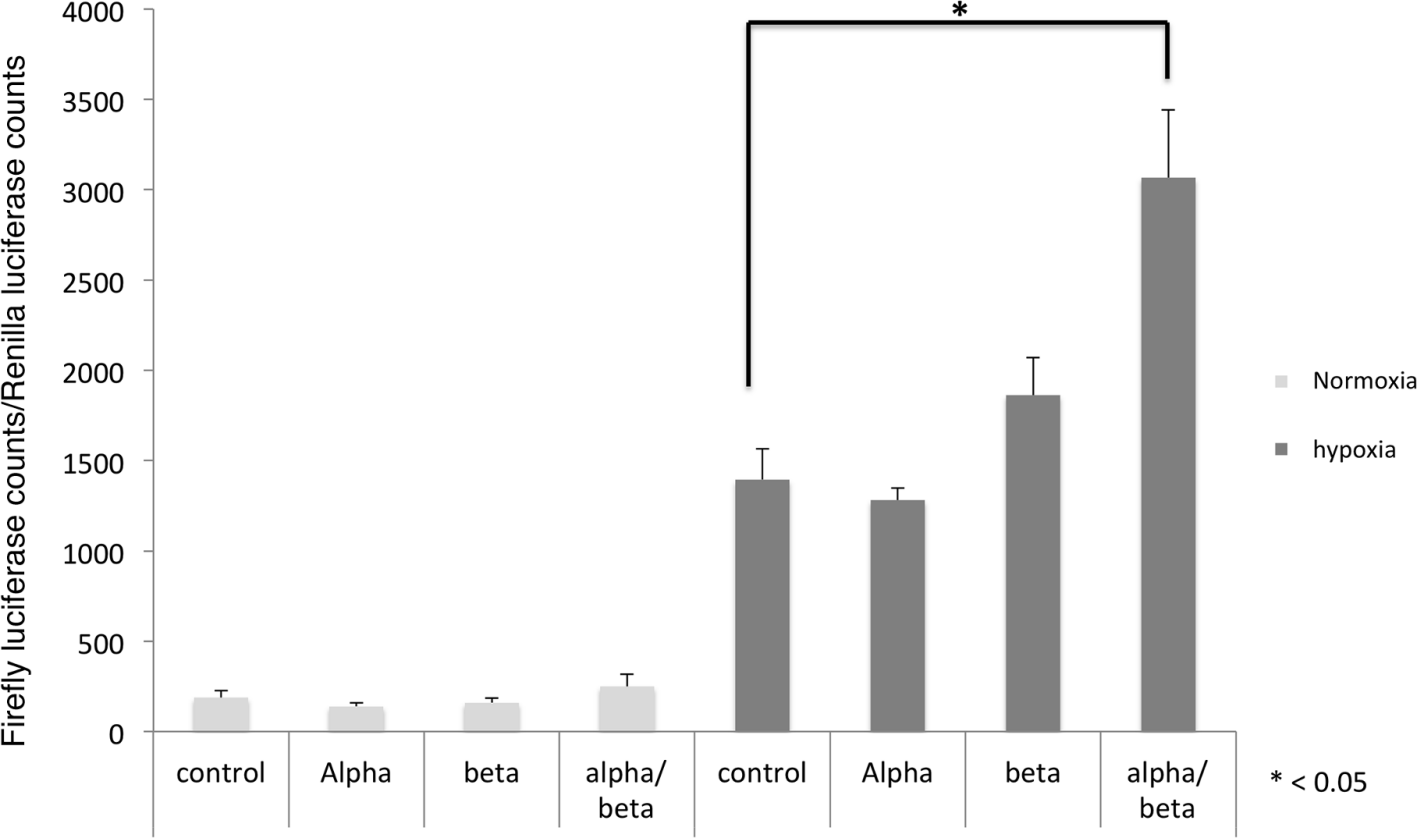
SpiHIF promotes human HRE transcriptional activation. HEK293 were transfected either with luciferase reporter plasmid together with empty, *spiHIF*α, *spiHIFβ*, or a combination of both *spiHIF* expression vectors. In all cases, a control Renilla expression vector was co-transfected to normalize for transfection efficiency. 24h after transfection, cells were maintained under normoxic or hypoxic conditions in an anaerobic workstation for 24h. Cells were lyzed and firefly and renilla luciferase activity were measured. A ratio of firefly/renilla luciferase activity is shown. Results are representative of three independent experiments performed in triplicate. Values are means ± SD (n = 3). * P< 0.05.

To characterize the functionality of SpiHIFα induced during hypoxia, we tested its ability to stimulate the transcription of a reporter gene under the control of Hypoxia Responsive Elements (HRE) [17]. In normoxia whatever the plasmids transfected, the luciferase activity was low (Fig 4). In hypoxic condition, the basal luciferase activity detected in extracts of cells transfected with the empty expression vector was more than 7-fold higher than in normoxic conditions. These results probably reflect the induction of the endogenous human HIF1 as previously described [23]. The luciferase counts were not statistically different in cells transfected with SpiHIFα or SpiHIFβ expression plasmids alone as compared with the empty-vector-transfected cells. When expression vectors coding for both subunits were transfected, luciferase counts were twice those measured in empty-vector-transfected cells (p < 0.05). These results strongly suggest that SpiHIF stimulates the transcription of a HRE-driven reporter gene in mammalian cells placed in hypoxic conditions.

## Discussion

The present study revealed that coral HIF shares several structural and functional similarities with human HIFs. Like human HIFs, SpiHIFα has two functional TADs (Fig 1). In the N-TAD, C-terminal ODD is present, but alignment of *Stylophora pistillata* and human HIFα showed a lack of the N-terminal ODD in SpiHIFα. Similarly to *Nematostella vectensis* [12], the analogous proline which is normally hydroxylated by PHD, was absent in *Stylophora pistillata*, in *Acropora digitifera* [24], and in all cnidarian transcriptomes published so far [25] (data.centrescientifique.mc/CSMdata-public_overview.html). A gene homologous to human *phd* is present in corals, suggesting that hydroxylation may occur on SpiHIFα putative ODD. However, mutation of P402 residue in human HIF-1α did not result in inhibition of HIF degradation, showing that HIF-1α may be degraded by a pVHL-dependent mechanism that is independent of PHDs [26]. Such mechanism might also exist in corals. Regarding the SpiHIFα C-TAD, it shares high sequence identity with the human HIF-1α/2α. A putative asparaginyl-hydroxylation site is also present in SpiHIFα. The presence of a *fih* homologue gene in *Stylophora Pistillata* suggests that SpiHIFα degradation may be controlled by this domain. These similarities and differencies between human and *Stylophora Pistillata* HIF, incited us to question HIFα regulation in corals. Our results showed that the coral HIFα is regulated by a similar mechanism as human HIF. When tested in human cells (Fig 2) or *in hospite* (Fig 3), hypoxia increased coral HIFα abundance. In addition, our results indicate that coral HIF stimulates a HRE-reporter gene expression in mammalian cells (Fig 4).

Although Scleractinia (corals) appeared ~250 million years ago [27], around the same time as the first mammals [28], these results suggest that coral HIFα can activate a HRE-dependent reporter gene expression in human cells despite 1200–1500 million years of separation in evolution between Radiata and Bilateria [29]. Altogether, these data suggest a strong evolutionary pressure of selection. for *hif* genes. However, the SpiHIF-mediated transactivation activity was low (about 2 fold). These results suggest that the coral HRE could be slightly different as compared to the human one, although the coral HIF bHLH region was able to bind the human HRE (Fig 3). SpiHIFα bHLH possesses only 51% identity with human HIF-1α bHLH (Fig 1A) and SpiHIFβ bHLH 51% identity with human HIF-1β bHLH. However, all the important amino-acids for HRE binding were conserved in both subunits [30, 31]. Further, SpiHIFα may not interact with coactivators such as human CBP/p300. This hypothesis is supported by structural analysis results (Fig 1A). In mammals, the C-TAD domain of HIFα is responsible for CBP/p300 binding. However, the percentage identity was only 53% between SpiHIFα C-TAD and human HIF-1α C-TAD and three (out of five) important amino acids for binding to CBP/p300 [32] were absent in SpiHIFα (Leu-795, Cys-800, and Pro-805 hHIF-1α notation). Substitution of these residues impairs human HIF-1 transcriptional activity [33]. However further studies are needed to demonstrate these hypotheses in corals.

The best alignment for SpiHIFα was obtained with the human HIF-3α. But SpiHIFα like human HIF-1α (but not HIF-2α nor HIF-3α) contained a conserved sequence for hydroxylation by FIH. Considering this observation, SpiHIFα seemed more similar to human HIF-1α. Consequently, despite these interesting observations, further experiments are needed to determine if SpiHIFα is more similar to human HIF-1α or HIF-3α.

In human, more than 1,000 genes are directly trans-activated by HIFs in response to hypoxia (for review [34]). Corals are well adapted to daily hypoxia and the next step will be to determine what genes are regulated by hypoxia in corals. Moreover, it would be interesting to investigate if there is a common set of genes responding to HIF trans-activation, despite the differences in anatomy/physiology between corals and mammals. Finally, assessing if corals possess a specific set of genes, possibly related to HIF, involved in main physiological changes would increase our understanding of coral metabolism Levy *et al*. have already suggested that the HIF system in corals mediates diel cycles in central metabolism [35]. Furthermore, diel changes in gene expression were evaluated by RNA-seq in corals [36, 37]. Both publications showed that about 450 genes are differently expressed between night and day, but none were investigated for their possible HIF regulation. In order to determine the genes regulated by HIF, the promoter of coral gene containing HREs must be cloned in reporter expression vectors since across the entire mammalian genome HIF binds to only a small proportion of all HREs [38]. Chip-seq experiments will serve to identify HIF binding sites across the coral genome

With growing concerns about global change, aside from temperature changes and ocean acidification, oxygen concentration in the ocean is of great interest. Indeed, the Intergovernmental Panel on Climate Change in its 2013 report considered that the oxygen decrease in oceans would be one of the major threats for coastal ecosystems. Regarding coral reefs, oxygen concentrations decreased since the 60’s in most tropical regions [39]. Moreover, during the same period, oxygen concentration has declined about 10 times faster in coastal ocean than in open ocean, and an increase in the number of hypoxic zones was observed [40]. This process could affect fundamental biological processes of the coral reefs in the near future. It is well documented that hypoxic conditions lead to mortality of coastal fishes and invertebrates [41, 42], but current understanding of how oxygen decrease can alter these ecosystems is poorly studied. Indeed, calcifiers such as corals are particularly sensitive to hypoxia [43] and decreases in coral photosynthesis under hypoxic conditions suggest that decreasing oxygen concentrations could have potential negative implications for reef ecosystems [44]. Recently, an unprecedented hypoxic event on the Caribbean coast of Panama has been described [45]. The event caused coral bleaching and massive mortality of corals and other reef-associated organisms, but not all coral species were equally sensitive to hypoxia. Further studies in different coral species might improve our understanding of reef resilience to increasing hypoxic conditions due to climate change. In conclusion, our study gives important tools to the scientific community in order to better understand the coral physiology and the impact of hypoxia in corals.

## Supporting information

S1 Fig Specificity of SpiHIFα antibody. HEK293 cells were transfected with 0.1μg of spiHIFα plasmid or empty vector. 24h post-transfection, cells were maintained in hypoxic (<1% O2) conditions during 6h. Whole cell extracts migrated within a 7.5% SDS PAGE gel and were analyzed by immuno- blot with anti-SpiHIFα antibodies.
(TIF)

S1 File cDNA and genomic sequence of SpiHIFα and SpiHIFβ. (DOCX)
