# Supplementary figures and images for: Structural and functional analysis of coral Hypoxia Inducible Factor
Source: PLoS One. 2017 Nov 8;12(11):e0186262. doi: 10.1371/journal.pone.0186262 (PMC5695583; doi:10.1371/journal.pone.0186262)

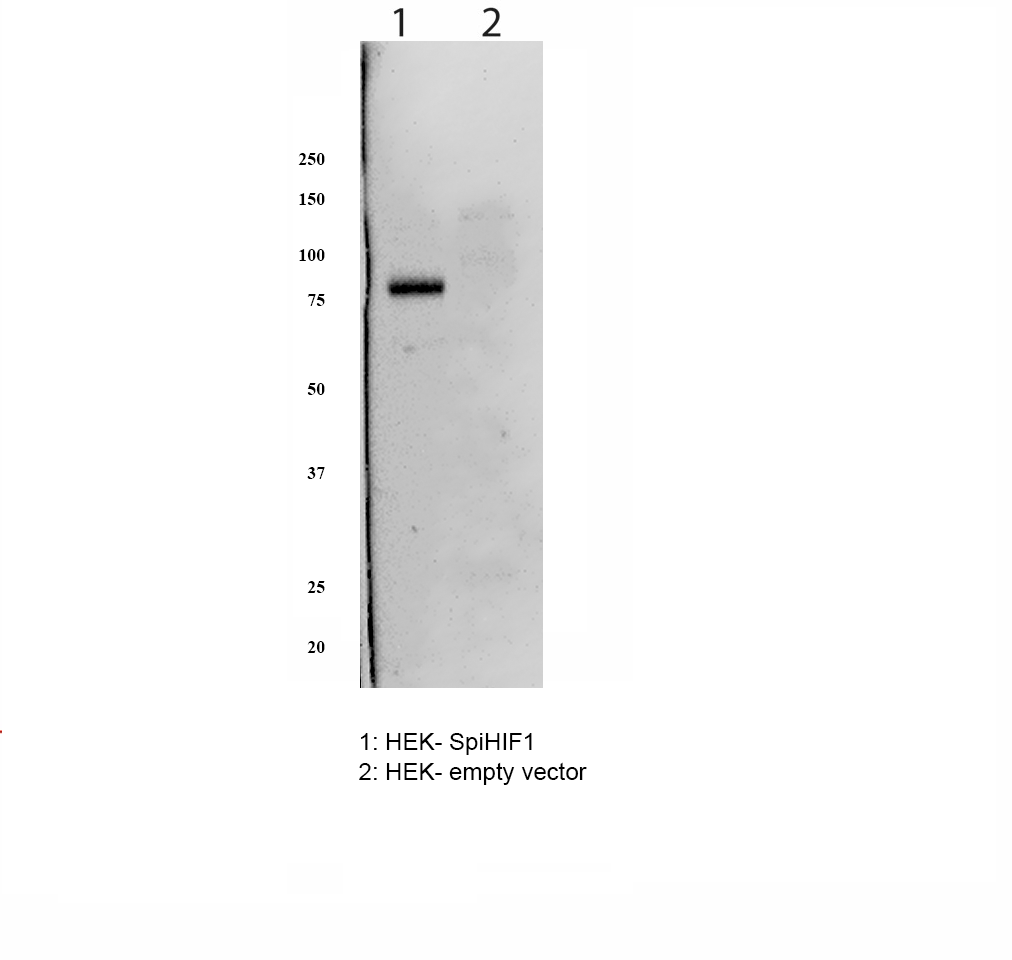

Supplement: S1 Fig — HEK293 cells were transfected with 0.1μg of spiHIFα plasmid or empty vector. 24h post-transfection, cells were maintained in hypoxic (<1% O2) conditions during 6h. Whole cell extracts migrated within a 7.5% SDS PAGE gel and were analyzed by immuno- blot with anti-SpiHIFα antibodies. (TIF) [file pone.0186262.s001.tif]
