# Supplementary material for: Structural and functional analysis of coral Hypoxia Inducible Factor
Source: PLoS One. 2017 Nov 8;12(11):e0186262. doi: 10.1371/journal.pone.0186262 (PMC5695583; doi:10.1371/journal.pone.0186262)
Supplement: S1 File — (DOCX) [file pone.0186262.s002.docx]

>SpiHIFα cDNA

GTTTTCAAGTTTTCAATACAGCAATGCCACTTCATATCTCATCCAAACACATCACGCTACCAAACGATTCGGCTCCACTGGAACAAATTCACGAAAGATCTAAACTAACAGGGATTTAGAACCCAACGCCGTATCTTTCCGCGGGGACGGATTCAACAAAAAGGTTTACGCCGTTAATTTTCCACCCTGGAACACCTTTAAGCAAAGAGGGATTACTTCACAATCCTCGACAGAGATTCGAGACTGTGGAGAAACCTTTGGAGCAACCAAATTCATCGAGAACAGATCACCGGACGAAGACAACAACAACATCCGTTCAGATAAAAATGGCGAGCAAGGCAGTAAAGACTAGTGAGAATCAGAAGAAGAGAAGATCACGTGATGCAGCTCGATCCAGGAGAGGACAGCAGAATGATGAGTTTGTGGAGCTTGCCAATCAGCTGCCATTGCCTGCAGGACTGTCATCGCAGCTTGACAGATTGTGCATCATGCGGCTGGTCAACAGCTACATTAAAATGAAAAACCTTCTTCAATCTCTGGTGTCCCAAGACACGAAGAAGATTGTCTCTCTTAATGTAGCAGATACCATGGTATACGACAAGGCGTCACTGGAGGCACTAGATGGGTTCGTCTTTGTTGTAACACCAGATGGCCAGTGCATTTATGTGTCAGATAACATAACCCATTATATGGGACTCACACAGATTGAAGTAACTGGTAACAGTTTCTACAAATATATCCATCCCTGCGATCATGAAGAACTGTCCAATCAGCTTGGAGGACAGATTCCACTGGAAGACATGGAAATCTTTGATGGCCTGTTCTGTTCGGATTCTGTCTTTATGATGAGCAACCACCTGAAGGGCGGATCAAAAAAATCCATCCATGAGAACCCACACAAGTCATTCTTCTTGCGCATAAAAAGCACATTGACGAGCAGAGGAAAGAATGTTAACCTTCGAGCCTCAACCTACAGAGTCGTCCACTGCACTGGTTGCATGAAAATGTCTGTGAAAGTGTCAGACTCAGGAGAAGAAGAAAAGGTTCCATTATTCATGGTCGCCATTGGAGTACCACTTATGTTCACATCCACTTTTGAAGTTCCGCTGGACAGAGCCACATTTACCAGTCGTCACAGCCTTGACATGAAATTCCTTTCATGTGATGACAGTGTATCAGACCTCCTTGGTTTCACACCAGCAGAGATTGTTGGCAAGTCTTGGTATCACTTCCAACATGCATGTGACTTGGACACAGCATTAGCATGTCACAAGACATTGCTGACCAAGGGGCAGTCTGTGAGCAAATACTACCGATTCTTGGTTCGTAATGGAGGATGGGTGTGGCTTCAGACCAAGGCCAACATTGTTTATGACAGTAAGACATGCCAGCCGCAGTTTGTCCTCTGCACAAATTACATCATCATGAGAGTTGATGAGGAGGATTTTGTACTGTCCACTGAACAACTCAACCCCATTCCTGCTCCTCTGGGTCTGGACCTTGTGGTGAAAAAAGAAAATGAAAAGGGAAGGGGAAGGGGAACTAGGAGAAAACATTCCATTGTGCTGGAGGAACGTACCAAGGTGCTGAAGGCAACAGCTAATGAACAGCGTCCAAGTGCAGACATCAAACCAAAGAAGGAGAGCAACCTAAAATCCTGCTGTATGAAGACACTCCCAAAACAGGCAGCAAAGAAATCCTGTCAGTGTCTTGAGGATGTTGACTGTGAGCATATTAATTTTCAGGAGTGCATTCCAGTGGCATGGGACACCAATGATGACCTCATTGAGCAACAAAAGGAATATGAAGAGCTGCTGCAAACACACTATGGTGTTACTGATGACTTTCCCAGTCCCACCCAGTCAGAAAACAGCACTGCTGAAAATAGAGTCCCCTACATTCCTTCTCCAAGTAACGACCAAAACATTGACAAAGGGAAGGTAGCTAATGAGGAGGAAGAAAATTTTGATGAACGTGCTCCATTTATTCCACTTTCTATCATGGACTCAGAGCTGGATTGTGATGATGATTTAATAGTTGAGATTCCTTTCCTTGATTCACCAATGATACCATCACCCTCATCATGGCTACTAAATGCTGACGAAAGCATTCCATCTCCAGGAAGTTCTGCTATGCTGCCTCCAGCCAACCCTCCACCCTACAACTGTCGCCCTAGTCTGTCAAACTCAAGCTCACCTGTGTTAAACAGAAAGGAGATCCCTATGGCTGTTAAACGCAATGGGTATGTGACAAACGAGACAGCTTACAAAGGGAAAGCACTGTTTCCAAACATCAGTCCCTGGGAGGCTGAAGTGAATGCACCAGTTCAGAATATTAATCTTTTGCAAGGTGAAGACCTGCTTGAAGCGCTTGAATGTGGCGACTTCTTGTGACCTTTGACTGATAGACTAAAGCCACCTGTATGTCTCATCTAGGAGTGACAGTCGGAGGACCTATTTCAATTATCTGTAGGTTATATACTCAACTTTACAAGTTGTTTGCATTGAATTTCTGATTGTATAGCAATATGCAATTCCAGTAATCTTCATCAGGGAACGTATGTCACTGATTCACTCATATTTAGCTTTAAGTTTGGAATTTTTTAGTTTGTTTTTCTGTTTGATGATAGAGTGGTGGAGTGGTGAGCATTATGGACTCCGAATAGAAAGGTTCGAGCCCTGGCCGAGTCCTTATGCATGACATGTTACCCTCCTACTGCCTCTCTCCACCCAGGGCAGGGTTGTTTAAAGCTGGGTTAAGATAACCTTGGGTTAGTGCGATATCTGGTTTCGGATCTGAAAGCTCTAAAGAAAATTTAGTATAATTCCTTTGAAGTACAATTCAATGATTGAATGCTCCAAAAAGAATAGAAAAAGTATCAGAAACAGCTTTTGAACAAAGGAATAACTTAAGAAACTCAGATTAAGAATCTGACGTTGGGTTTGGGCTAATCAGCCTTTGAACACCTGGGCCTGCAGAAATAGAAATGGGTACCAGTTAGCTTTCAGGGAAACCCAAAATAATTGAGGAGGACTGGGGAATAGCTGTTATGGACTGTCATCCTATCCCATGTAGATATACAACTAGAAATATGTGCAAATCAGGGATAAGCAGCCACAGGCCACAGTTTTGAGCTTCTTGGTCCTAAGATTTAACAATACCTTTTTTTCCTTAATAGGGAAATATGAATAAGCAATAAAGTTACCTGTACTGATGTGGTGTAGACAAACTTTTTGCAAGTTGATTGTGTTGTGTTTGCCACTCAACAGATTTTTGACATGTTTTCCAAATGTGAGGCACACTAGAAGGCAACTTGACAGTATATAACAATATTAAAAAATTGTCTCAAGTTTAGGAAAAAGATATCTTTAGTGAGTTCTCATTCAGAAATTTATGCCTGCTGCATTTGCAGAGAAGGTGTCTAAAACCTTGTTGAGTGGCAGGGTAACAATTATCACTACCAGTTTTTGATTCTGGTGAAAAAGAAGCATACTGAGTTAAATCATTCTTCAAAGAGCAAAATCTTAAGCTGTGCCTCACAGATACCAAGGCAGTAAATGCAGAAAATCCTAGGAGCAGGACTTATTTCTCAACTGCAGTTGGTTATCAAGTTTAAAATTGTCCAGACTGTTTGGATTCAAACAAAAGAACTTCTAGCTTCACTCAGCAGGTTTAATGACACTAAGATTGTCCTCGTAGAGTGGTAGTTTAAATTGTGTGTATACATTCAGTCACAACAGTATTAGTCCAAAGAAAACATAGTATTTTATAGTAGCTTCAAAATTAGTTGTAGTATACTACAAGATTTTACTCATATAGTGCAGTATTGAATGTAGTGTATGTCTATGGCTGAGTGCATTGTGTGCCTCCTTTGTTTTCTTTGTGGTGGTGTGAATGTTTGTCCTACTGATCACTTGATGGCTTCTTTGTGCCTGTGTCAGTTTGGTCATTTGGGAATGGAATGTTCTAAAGGGAATCAAATGTTATAAAGATCTGCATTAGGAATTTTGTAACTTTACAGTGTAGTGTGTTTGAATGCTCTTGTTGGTCTGCTAGTCTAACTTGCAGAGGTGTGTGGAAAGCCTTCAATGTAGAATCACAATTTATTTCACCAATAGTGATGCATATAAACTTAAAAAAAAAAGTATTGAGTGATTGATATAGCTACAGGCATATTTAGTAAGGTTGTATTGTTGGCAAAAACTGACAACTTGAGTAAACTTCTCAAGAGCTTTACATCTTTGAATTCTTCATCATTAAAATCAAAACATGTACAGGTATAGATGCTGGTCAATGATCTACAGGATGAGGAACTCTGAAATCCTTTCCCATAAATGAGTAGGAAAGTTGCTAGTTCAGAAGTGAAATCTTTTGATCCCTTATAACCATTTTTTCTTCATTGAAGCTCTCATTTGGGAACTCAACAGTTTCAATAAATCATCTCTTTTGCACAAAACCCTGACATATCTTCCCTCAATCCTGTTGTAGGAGTTCAAACAAGATCACATAGATCCCGTGGAAAATTCATTCTGGCCTGGGTTTGTTGCCTTTTACATGATCATTAAAACTTCATTGCTTTACAATCATTTTATCTGACCAACTTAAAATCATGTGTGAGAAAGTAATTCCTATTCAGAAAACAACTGAAATACCATTAAAAAGATTCTGAACTGGACTGTTGGGCTTTCTTTGAGCTCAAAATACTTGGGCTGTCGGGCAGTTTGTGGGAAATTTCAGCAAAATCAATCCATCTCTCATCAATCACACAGCTCCAACCTGCAACAATTTTCCCATCTTGTCTAGAAACCTCCATAGCTCTGCCCTTTTCAAACACTGGTTTGGAAAACTAACAAAGTTCTATGCCCTATAATGAACCCTATGTGTAGTTGTAGAAATTCGGCCTGAGAAAAAAAAAGGAAAAAAAAATTCAGGGCCATACAGGATTTGAACCCATGACCTCTGCGATACCGGTGCAGTGCTCTACCAACTGAGCTAACAAGCCAACTGGGAGTAGGCCGTTAAGTTCGAATCCCATATAGGCCTGATTTTTTTTCGGGTCTGATTTTCGCTGTTGCTTGGATGGCGC

>Genome scaffold containing the SpiHIFα 

GTATAATGAATTTGATTGTGTGACTCTGCTAGCTATGTTCTAATTGTCTTTGTTACAAGTTCTTGAACACCTTCCTCGCAGTTTTTGCAATCAAAGTCTTTTCAACAGTTAGGTTCCCTGAACTTTTCTAAAAACATTTATTGCAGATAGCTTCATTACAAGGTGGTTACTATAATTCTTCTGTTGTTCACATGTAGGTAGACTACATTGTAACACCCACAAGGATTATCCAATGCCAAGGACATAAACAGAGCCCAGTTGGAATACAGTGGTCAAAAGTCACACCTCAAATGTTGAAAGATATTCCTGTATTGAAAGATCTTAGAGAAAAAGAGAGAAGAGTTGGAAAGAATGTCACTTTGAATGAGTGACTTATTGACAAGATTCTGTTGATAATTGGGTTCCTTGGATAACCTCAATAATGTTTCTTTTTTATATGATTCATTTAGTTTGTAAGAACATGTGACATATGTGACTATGCCTTATGTGCCACTTGTTAAATGTTCTTAGTGCATTTTTACATCATCTGTGATTTGTTACTGAACAGATGCATAGCACTGCACCAGTATTGCAGAGGTCATGGGTTCAAATCCTGTATGGCCCTGAATTTTTTTTTCCTTTTTTTTTCTCAGGCCGAATTTCTACAACTACACATAGGGTTCATTATAGGGCATAGAACTTTGTTAGTTTTCCAAACCAGTGTTTGAAAAGGGCAGAGCTATGGAGGTTTCTAGACAAGATGGGAAAATTGTTGCAGGTTGGAGCTGTGTGATTGATGAGAGATGGATTGATTTTGCTGAAATTTCCCACAAACTGCCCGACAGCCCAAGTATTTTGAGCTCAAAGAAAGCCCAACAGTCCAGTTCAGAATCTTTTTAATGGTATTTCAGTTGTTTTCCGAATAGGAATTACTTTCTCACACATCATTTTAAGTTGATCAGATAAAATGATTGTAAAGCAATGAAGTTTTAATGATCATGTAAAAGGCAACAAACCCAGGCGAGAATGAATTTTCCACGGGATCTATGTGATCTTGTTTGAACTCCTACAACAGGATTGAGGGAAGATATGTCAGGGTTTTGTGCAAAAGAGATGATTTATTGAAACTGTTGAGTTCCCAAATGAGAGCTTCAATGAAGAAAAAATGGTTATAAGGGATCAAAAGATTTCACTTCTGAACTAGCAACTTTCCTACTCATTTATGGGAAAGGATTTCAGAGTTCCTCATCCTGTAGATCATTGACCAGCATCTATACCTGTACATGTTTTGATTTTAATGATGAAGAATTCAAAGATGTAAAGCTCTTGAGAAGTTTACTCAAGTTGTCAGTTTTTGCCAACAATACAACCTTACTAAATATGCCTGTAGCTATATCAATCACTCAATACTTTTTTTTTTAAGTTTATATGCATCACTATTGGTGAAATAAATTGTGATTCTACATTGAAGGCTTTCCACACACCTCTGCAAGTTAGACTAGCAGACCAACAAGAGCATTCAAACACACTACACTGTAAAGTTACAAAATTCCTAATGCAGATCTTTATAACATTTGATTCCCTTTAGAACATTCCATTCCCAAATGACCAAACTGACACAGGCACAAAGAAGCCATCAAGTGATCAGTAGGACAAACATTCACACCACCACAAAGAAAACAAAGGAGGCACACAATGCACTCAGCCATAGACATACACTACATTCAATACTGCACTATATGAGTAAAATCTTGTAGTATACTACAACTAATTTTGAAGCTACTATAAAATACTATGTTTTCTTTGGACTAATACTGTTGTGACTGAATGTATACACACAATTTAAACTACCACTCTACGAGGACAATCTTAGTGTCATTAAACCTGCTGAGTGAAGCTACAAGTTCTTTTGTTTGAATCCAAACAGTCTGGACAATTTTAAACTTGATAACCAACTGCAGTTGAGAAATAAGTCCTGCTCCTAGGATTTTCTGCATTTACTGCCTTGGTATCTGTGAGGCACAGCTTAAGATTTTGCTCTTTGAAGAATGATTTAACTCAGTATGCTTCTTTTTCACCAGAATCAAAAACTGGTAGTGATAATTGTTACCCTGCCACTCAACAAGGTTTTAGACACCTTCTTTGCAAATGCAGCAGGCATAAATTTCTGAATGAGAACTCACTAAAGATATCTTTTTCCTAAACTTGAGACAATTTTTTAATATTGTTATATACTGTCAAGTTGCCTTCTAGTGTGCCTCACATTTAAAAAACATGTCAAAAATCTGTTGAGTGGCAAACACAACACAATCAACTTGCAAAAAGTTTGTCTACACCACATCAGTACAGGTAACTTTATTGCTTATTCATATTTCCCTATTAAGGAAAAAAAGGTATTCTTAAATCTTAAGACCAAGAAGCTCAAAACTGTGGCCTGTGGTTGCTTATCCCTGATTTGTACATATTTCTAGTTGTATATCTACATGGGATAGGATGACAGTCCATAACAGCTATTCCCCAGTCCTCCTCAGTTATTTTGGATTTCCCTGAAAGCTAACTGGTACCCATTTCTATTTCTGCAGGCCCAGGTGTTCAAAGGCTGATTAGCCCAAACCCAACGTCAGATTCTTAATCTGAGTTTCTTAAGTTATTCCTTTGTTCAAAAGCTGTTTCTGATACTTTTTCTATTCTTTTTGGAGCATTCAATCATTGAATTGTACTTCAAAGGAATTATACTAAATTTTCTTTAGAGCTTTCAGATCCGAAACCAGATATCGCACTAACCCAAGGTTATCTTAACCCAGCTTTAAACAACCCTGCCCTGGGTGGAGAGAGGCAGTAGGAGGGTAACATGTCATGCATGAGGACTCGGCCAGGGCTCTAACCTTTCTATTCGGAGTCCATAACGCTCACCACTCCACCACTCTATCATCAAACAGAAAAACAAACTAAAAAATTCCAAACTTAAAGCTAAATATGAGTGAATCAGTGACATACGTTCCCTGATGAAGATTACTGGAATTGCATATTGCTATACAATCAGAAATTCAATGCAAACAACTTGTAAAGTTGAGTATATAACCTACAGATAATTGAAATAGGTCCTCCGACTGTCACTCCTAGATGGGACATACAGGCGGCTTTAGTCTATCAGTCAAAGGTCACAAGAAGTCGCCACATTCAAGCGCTTCAAGCAGGTCTTCACCTTGCAAAAGATTAATATTCTGAACTGGTGCATTCACTTCAGCCTCCCAGGGACTGATGTTTGGAAACAGTGCTTTCCCTTTGTAAGCTGTCTCGTTTGTCACATACCCATTGCGTTTAACAGCCATAGGGATCTCCTTTCTGTTTAACACAGGTGAGCTTGAGTTTGACAGACTAGGGCGACAGTTGTAGGGTGGAGGGTTGGCTGGAGGCAGCATAACAGAACTTCCTGGAGATGGAATGCTTTCGTCAGCATTTAGTAGCCATGATGAGGGTGATGGTATCATTGGTGAATCAAGGAAAGGAATCTCAACTATTAAATTATCATCACAATCCAGCTCTGAGTCCATGATAGAAAGTGGAATAAATGGAGCACGTTCATCAAAATTTTCTTCCTCCTCATTAGCTACCTTCCCTTTGTCAATGTTTTGGTCGTTACTTGGAGAAGGAATGTAGGGGACTCTATTTTCAGCAGTGCTGTTTTCTGACTGGGTGGGACTGGGAAAGTCATCAGTAACACCATAGTGTGTTTGCAGCAGCTCTTCATATTCCTTTTGTTGCTCAATGAGGTCATCATTGGTGTCCCATGCCACTGGAATGCACTCCTGACAACCACAAAAAAATAAATATTAATAAATACATCATAGATCATATTTGGTAAGATTGCCTAAAAAAGTACTAAGGTTTTAAAGTACACAGTTAAAAATTCACTTTCTAAAGGCCTCCTGGGTTTAATTGACTCAATATATAACAAGAAATGATGAACAATTGTGCACCTTCTAGGCCGAGCTCACCTGAAAATTAATATGCTCACAGTCAACATCCTCAAGACACTGACAGGATTTCTTTGCTGCCTGTTTTGGGAGTGTCTTCATACAGCAGGATTTTAGGTTGCTCTCCTTCTTTGGTTTGATGTCTGCACTTGGACGCTGTTCATTAGCTGTTGCCTTCAGCACCTTGGTACGTTCCTCCAGCACAATGGAATGTTTTCTCCTAGTTCCCCTTCCCCTTCCCTTTTCATTTTCTTTTTTCACCACAAGGTCCAGACCCAGAGGAGCAGGAATGGGGTTGAGTTGTTCAGTGGACAGTACAAAATCCTTCTCATCAACTCTCCTGAGGAAATAAGTTCAGATAACAATTCATTAGTCCTAGTAAACTTCTCTCCTGTTATACTGAGGGAGATGTCCCTTTTGCAGTGTTTTATTTTATGAGGGGTACAAGTTCGAAATTGAATCAAAAGTCCCCTAAATTTACCAGACATTTCCCAGAAGGGCGAGCTTGGTGAACAGGTCTAAGGATTTAGTCATACATCTGTTAAGATCAAAGAGACACTACTCATGGATGAACTTGATAATAGATACAGACATTTTTTTTTAACAAATATCTCCCAAACATGACAACACAGTTTTCCTGTGAGTACATAAGAAATCTTCCAAACCACTCCCTTTCACTCTGAGTACACCCAGTCTTTCAGCTAAACCACACTTCTACTATGTTTTTGAGCTACTGTACATGTAAATTATTACTATTCCTTTTAAAATGTGTCTATAACAACTTTAAAATCACTCCTGTTTTAAACTTACATGATGATGTAATTTGTGCAGAGGACAAACTGCGGCTGGCATGTCTTACTGTCATAAACAATGTTGGCCTTGGTCTGAAGCCACACCCATCCTCCATTACGAACCAAGAATCGGTAGTATTTGCTCACAGACTGCCCCTTGGTCAGCACTGTGANNNNNNNNNNNNNNNNNNNNNNNNNNNNNNNNNNNNNNNNNNNNNNNNNNNNNNNNNNNNNNNNNNNNNNNNNNNNNNNNNNNNNNNNNNNNNNNNNNNNNNNNNNNNNNNNNNNNNNNNNNNNNNNNNNNNNNNNNNNNNNNNNNNNNNNNNNNNNNNNNNNNNNNNNNNNNNNNNNNNNNNNNNNNNNNNNNNNNNNNNNNNNNNNNNNNNNNNNNNNNNNNNNNNNNNNNNNNNNNNNNNNNNNNNNNNNNNNNNNNNNNNNNNNNNNNNNNNNNNNNNNNNNNNNNNNNNNNNNNNNNNNNNNNNNNNNNNNNNNNNNNNNNNNNNNNNNNNNNNNNNNNNNNNNNNNNNNNNNNNNNNNNNNNNNNNNNNNNNNNNNNNNNNNNNNNNNNNNNNNNNNNNNNNNNNNNNNNNNNNNNNNNNNNNNNNNNNNNNNNNNNNNNNNNNNNNNNNNNNNNNNNNNNNNNNNNNNNNNNNNNNNNNNNNNNGTTACTCACATGTCTTGTGACATGCTAATGCTGTGTCCAAGTCACATGCATGTTGGAAGTGATACCAAGACTTGCCAACAATCTCTGCTGGTGTGAAACCAAGGAGGTCTGATACACTGGGGGAAAATAAAAAACAAGACATTGACTAAGTGAAAACACCAGAGCGTGCATGAGGGCTAAAAAAAGGCAGTAACTTTCTAGACTTGTATCTGGAAAATTGCCACAGTTTCACTCAATATTATCCAGTGTGGTGAACTACTTCATTGGCCCCACAAATTGAGAGTTCACCAGAAGATGACACCTTAATTTTTCTTTTTCCAGTAAACCAATAATAGTGAAAACTAAAATAAAGGAGTTTCCCTTTAAACATAAAAATTGATCTATGATGTTGGATCAGGGTGAGATTAAGTTTCTTCTGCAAAGAACAAGTTTGTAAATGAGGCAAGTTTGGGAAGAGGAAAATATTTGAAAAACTGCAAAAGCCACGCGATAGAAGGAGTTCATTCACCAACCTGTCATCACATGAAAGGAATTTCATGTCAAGGCTGTGACGACTGGTAAATGTGGCTCTGTCCAGCGGAACTTCAAAAGTGGATGTGAACATAAGTGGTACTCCAATGGCGACCATGAATAATGGAACCTTTTCTTCTTCTCCTGAGTCTGACACTTTCACAGACATTTTCATGCAACCAGTGCAGTGGACGACCTGAAGGAACAAAAAGAACATTTTAACCCATTAACTTCCACGAGTGACCAAGTCAGACTTTCTCCTTATCAAGCAGACAAGTGATGAGAATAAAAAAAAATATATATATTAATGTTAGGATTATTGGCATGATAAGAACTGCATAGCAGACAGTAAGGAGAATTGCTAACCAGAATTTTGGGTATGAAAGGGTTAACAAATTGACATTTTAACATAAGAATGATTTTGGTGACATAAATCATAAGTTTTCAAGTGATTAACCCCTAAAATGAAAGTCATAGAGTAGCATTTTTGTGTTGCGCTGTCTGTAGTGATGTTGAAGACTTTTGATTCTGCAGTTAAAACCCTTGTGTGTGCCCATTCAAATGAAAGGTACTGAGCAGTACTTTGGTGTGGTGGTGTTTATTTAATCTAACCCTTCACTTTGATAATCAATTTGATGTTGGCCTTACTCTGTAGGTTGAGGCTCGAAGGTTAACATTCTTTCCTCTGCTCGTCAATGTGCTTTTTATGCGCAAGAAGAATGACTTGTGTGGGTTCTCATGGATGGATTTTTTTGATCCGCCCTTCAGGTGGTTGCTCATCATAAAGACAGAATCAGAGCAGAACAGGCCATCAAAGATTTCCATGTCTTCCAGTGGAATCTGTCCTCCGAGCTGATTGGACAGTTCTTCATGATCACAGGGGTGGATATATTTGTAGAAACTGTTACCAGTTACTTCAATCTGTCAGAGACAAAAATAACAATGCAATCATATTTAAGTCTTTTATAATATTGACGCCAATTAGGAAGGTTAATTGTTGGGAATAGTAAACCCTATAACTCCCAGAAGTGATTAACATGTAACTTCTCCCTATGACATTCATACATTATCAAGCAAAGAGGTGGTGAGAATTTTTATGTTATCAGGTGGAAGTTGAATGTTTTATCTTGATCTAACAACAAATTCTCACAATTAATTTACAGAGAAATGTATAGCAGCTAGAGGGGAGAATAAGCAATCAGATCATGGGCTAATTGGGAGTTAAGTGACTATTACCTCTACCTTTACCCCAATAGAAAAGAAGATCAGTGGCCATTAGCCGTTTGTCAGAGAAAAAGGACAGAGGGCTAACAGACTGTGAGGAAGGGCTAACACTCAAAATGTAAAAGGTGGCCAATTTACATTACTCACTCAGTTGATAAAACCAAATTACCTTGTATCTCCATAATAACTCATTAGGCAGTAGAAAGATTATAAAAATAGACAGTCATTTGCTGGTGAATGCTCTGACCTTGATACCACTCAACTTCTCTTTAGCTGATTAATAAGGAAATGTATTGTGGCTAGAGGAGAGAATTTGGAATAAGATCTTGGCAGTTTCAGAGGGATTGGGTGATTTACCTGTGTGAGTCCCATATAATGGGTTATGTTATCTGACACATAAATGCACTGGCCATCTGGTGTTACAACAAAGACGAACCCATCTAGTGCCTAAAGACAAACAAAATATGGTAAATTTCTGAGTACCAGAGTGTTAATTCTCCTTGTTCACTATCAGATAAAAAGTCATAACACATTTTGAGGCTTATTACTGCTAATTTAAAGCTCGTCTGTAGCATGAGATAGTGAGGTGTTTAAACAGTGTAAATTAAAGCTTACCTCCAGTGACGCCTTGTCGTATACCATGGTATCTGCTACATTAAGAGAGACAATCTTCTTCGTGTCTAAAAGAATTTATAAGGGTAATTAGTATAAGATAAAAAAATTTTTCAAACTGTTTGAATCTGTAAGGAAAAAGTCCTTACCTTGGGACACCAGAGATTGAAGAAGGTTTTTCATTTTAATGTAGCTGTTGACCAGCCGCATGATGCACAATCTGTCAAGCTGCGATGACAGTCCTGCAGGCAATGGCAGCTGATTGGCAAGCTCCACAAACTCATCATTCTGCTGTCCTCTCCTGGATCGAGCTGCATCACGTGATCTTCTCTTCTTCTGATTCTCACTGTTTATGGATAAGATAAGGAGATCAACCCTTCAGAAGTGATTAAAATTTAACTTCTCCCTATAATATCCATACATTATCCAGTAAACAGGTAATGAGAATACTCAAACTTAACAGTTATCAAGTTTTTATCTTGCTCAGGTACATGTAATCTCAGCAAGAATGGACCGAGATTTTGGGCTGCACACTGCTTTGTTGATAACCGTCTTCAGAAATGTTTTTAAATTCTAGATGTTTCTCTGCTGTTCACTTAATTTATAGATATGAAAATCACTAACATTTGAGGCCAGCAAACGAGTGTGGCATATATGGGTACAATTTGATGTCACACGATTGCAGCAAAATAGTTTTTTTTTTTTTGGCAGATTCTCTAAATTGGTACCGCTAACAAAAATGGCCTATTTGCAGATTGCAAAATATAGTAAAAAAAAAATATAGTAATGAATTAACTCTTGTGAAACATAAATAGCAGCTGGCAGATATTTAAATGTCAGCATCACAGAAATAGCTCAGAAAAAGACAATTTTAGCAAGAGAATGTAAATGAAAAACACAAGAGACCATATGTGGGCATTTATCATGGTTTACCGATAAACAACAATGTAAATATTTGCTTCACCAAGTCTACCTAATTCTGATGCAATTACTGAAAGAAAAGGGAAAAAAAACTTGATACAACCTTCTGAAACGCTCTTAAACGAACAATATTTGCACAGTAAACGTTTGCGAGCAAATAAATGCAGATCAAACGAGTAATTACAAACCACATCACACCAATCAAACCGCTGATTGTGACAAACAAAAACTAGAATTACTGAATTCTACCTTAATCGTGTACACACGACGTTTAATAAACTTCATATCAAACTATTATTTCCTCGATTTTATCTGCAAACGAAAACACGCTGGTTTCGTCAGTGGAAAATATAAGCATTTAGTGCAGTATGTGAAAGCACAATCGCTTAAGTGTTATTAAAGGCCTTGAAAGAGAAGCAATGGCGAGAAACATTTGCAAGTTTTGAAAGAGGAGATCGCGTATTATATTAGCATAACAGATATCGCAATCGACTCACATAATACAAGAAAATGAACTTTGGAAACTCTCACTGATACAAAAAAAGACGCTGCAAAGATTGTACTAAATAAGCTCGATAGCGTGTTTGGTACAAGCAAGGAAAACTTCAAGGTGTCATACTAACTGACGATCAATCTTCGAGGTGTCAGGCGCCTGGAGTGAATAATGACTTAGGTTTGCTCACGAATGTGTACAAAAGATTTTTTATTTCTAAACAATTCAAATCCTAACTATTCCCTCGAGAGTCGCAATTTGCAATGCGTGATGGCTAATTCTAATTTAAAGACAAAGAAATCGCAGACAAAACGTACCTAGTCTTTACTGCCTTGCTCGCCATTTTTATCTGAACGGATGTTGTTGTTGTCTTCGTCCGGTGATCTGTTCTCGATGAATTTGGTTGCTCCAAAGGTTTCTCCACAGTCTCGAATCTCTGTCGAGGATTGTGAAGTAATCCCTCTTTGCTTAAAGGTGTTCCAGGGTGGAAAATTAACGGCGTAAACCTTTTTGTTGAATCCGTCCCCGCGGAAAGATACGGCGTTGGGTTCCTACGAAATCGAGGCACGATATTGGTTTACAATCTCCAACACTGCAAAATTACAACCTCTATTTTTCATCCGAAGCAAATTACAGAGCTATGATAAGAAAACCGTTCTCAAAATAGCGAAAGAATGACGGGACACTTACTAAATCCCTGTTGGTTTAGATCTTTTGTGAATTTATTCCAGTGGAGTCGAATCGTTTGGTAGCGTGATGTGTTTGGATGAGATATGAAGTGGCATTGCTGTATTGAAAACTTGAAAACGTGCTTAGAATATATTTTAATAAATTAGCATAGTAATACTATTGTATCCGGGTCAAAGTTGTTTTTTTAATTCCCTTTTGAAAGGCATTTATGGTATTTACCTTATAAATGTGAAAGAGGCAGATTTATTTGACATTCTATCATGAAATACAAGAAAAGAAGCGAAAATACATCTCAACAGCCCCAAATATTTTTCCCTCTTTTCTTTTAATTTTCAACAACTAATGGTTTGTTAAAGATTTCGGAAGTCATTCATAGATTGTAAATGAGATTATGACGTCAGTTGACAGACCGTGTCCGTAGCCCATTTGATACGTGAAGATGACTAAGTAATCTATTTGACCTATTTCACACTTTGCTGCTTTTTTAAATTGAATTGAGATAACAGTTGTACAAATTGTGGGAATTTTCACAAAATAGAACAAAGAATGCTGCACGAAACTTAGATATGACTTCTGACAACCCAGTATCGAATGAAACGAGCGCAAAACAGGTCCTTCAATCTTCGCATATATTTTCCAGATTAAGCCAGATGCATATCTGCTCCGTGTCGGACCAATCAGAGCGGCGTGTTTAGAACTATGACGTCAAATAATTTTTTTACGTTAGTCTCGCAGTGTTTTCGTGGCCAAGGCCACTGGGGCGGGTACAAGCGGTTGAGACTGCCCAAACTTGGTTTTACATGTACCGAGGCTATGGAATTCCAACGCGTGCTTTAATGATCCATTTCATTTCATTTCATTTCAATAATTAAAACTGTTTATCAAGGCGCCATGGTGACAATTTACATTGTTTGCCCGGTGTAAGGAGATGATGCCAGAAACACTTTGTACAATACGTTGCTGGGGAAAGAAGTAGAAAGAGATACTCTTTGTAGTAAAAAAAAAATCTTGTTACACACCACGATTTCTTTCTTCATTCTAATATCACCTTTAAGGCGTAGTTGCTCTTAATCTTCATTTCATTATTATTGTAATTATTTATTATTACTAATTCTATAATTTCTTCCGAAGTCTTGTGAATTTCAACTTTCATAGGACAAATGCCTTTAGTGAAAGGAATCGAATAGATTGGATGTTTGTTTTCACACTCAATTTCCGCACGTGACTAAAGGTGGGTGCAATTTCCGCACGTGACAAAATTTGACTGTTGAGTACTCTCGCGTGATGATTCTCACGTGCGGCACGTGAGGTGTTTTTCTCAGCGAAGTGTAATGGGATATGTCAGTAAACAAGATTTAGATGGTGAAATCTGAGCTCGCTCTTGAAGTAGGGAACCCAGGTCAAGGGGGGAATTTTTCAATAAAGACACATTTTCTCTGAACCACGATCGTGACAAGAGCTTACTTGGACCCAATGGAAACTTGCCAATTTGTCTCTTGATCTGATTTGATGCTCGTTCATATAACAGGCTCTCATGATGCCTTCCCCTTGTAGCGTTCGTGGTTCGTACTCTTTTCTCCTGTAAAGTTTTTATTATACTGCAAACTATTAATAAGATTTATGCTGAGCATTCACTCACAAATATAAAACAATCGCGAAACTTTCACGTGGGTGAAAATTGATTTCTTAAATGCATATTGTGATGCACACAGGAAATTTATACCAGCGCCAAAGTAAACCCTCGATTTGGTCATTTCAGATAGATTCACTTAGCAGCGAGTGGGTTATACACCTGCTTAAGGAGTGTAAGAAAAATTGCTGGGGTCTTTGCCATCAAGTTGAGGAGGGGTCCATTGTAACTGACAATTTGCATTATCTGAATGTAAATTAAATGTGGGAAGCAATGTGGAATTACAAGAAGCAGAGTTAATTAGGCGTGAAGTTTCTGGAAGCTTGACAGATTTCGTTATAAAAGGTTTTTTTTAAGGATTTAAGGACTGGGCGAGTAAAATGTCCACAAACATCGATGTACAGTGGAACCTCGATTTAACGAAGTGCTAAGGGACTGGGGAAACGGGTTCGTTATATCGAGGGTTCGTTATCTCGAGGTTTTGTGGAATACATTTTACGGTAACTTAAGCTGGGCTGAAACTGAAGTATCGTTCGTTATAGTTCGAGGACCTCGTTTTTTAGAGGTTTGTTAAATCGAGGTTTCACCGTAATGTTTTTTGACCAATAGAGAAATTCCTTCAAATTAATCAATCACTGAGCGAGATCAAGAAATAAAATTTTGCCAGTGCACTGTGAAGGAGCTCTTAGCCTAACCTACAGCTCTATTGCTAAATTTTTTAATTTTACCGGCTTTCAGAACAGTTCTTTAGTACTAACCTTGTAAGAGGCTTGCATACGTACCTTGGGTGCAGAAACACCTGAATAAAATTGTGGTAAGTACGCTAGCACTTGACCTAGCGATGCTGGTTCACAGTTTTAATCATAGTCAAACTTCCTGTTTTGAAGCAAGGTCCATTAGATATATCGTATTATTTACGCCCTCACGTACTTTAGACGGGGTGGAACTTAAAATATAATATCTGTAATTCTTCGCACAACTGCAGATTTGCGCTGAACCGCACGTTTTTTGATTTCTGTAGTTAGCATAATATTAATGCATTGTTGCCTTGACCTGGGCTCGAGATGAGGTCACGAGACAAAAACCTTTTTTTTTCTTTTTTAGTTTCATTTGTTAGTTTCATTTTTCTGAGTTTACGCTGTCTGATAGAAAGGGAATTTAATCATGAATTTCGGTGATCACTTCAAAAATTCGAACCACTGATCGAGTCAGCTTCATCGAACAAGAAATCGAAGTAAGTTTCTCAACCCCTGTAGCTGTCTGTCATTTAAAAACCGCACGATGTGACAGCGCAAAAAGTTTGTCATAAAACAATGGACCTCACGTACGTGATGTGCAGAAAAATACCTTCACGTCTGAACGCTATTGATCTGTTATTGTAAATAGCGCTATATAAGAAATAAATTATTATTATTATTAACAAGAACTTCATGTATTTCTGCGCATTTGATCGTATTTTAATGTTAATTTGCGCGCTATAAGTAATAAAATTATTAAAAACTATTATTAAATAATTCCTCAGTAGCGCCAAGCGCTGTCAGTTCTAACTACATTTAAACTTAACAAACTATATTTGTGTTAAAGCTAAGCTTAAGAAATTTATGAATTTTTCGATACATGTCGGGTAAACACAAGATAGTAGAATTTTCCGTAATAATTAATTAAAAAAAAAAAAATTTAACAACTGAATTCTGCAAAATGCCTATTCTAATTTCGTTGCAAATATTGTGGCCACCAGACCCTATTCAAGAGCTAAGCATCTATCGAGTCTGAATTATTAATTGATTCTCAATATTTATTTCTCGATATCTCGGCTCTAATTTACATTGTAGAGTTAAAATTTAGACCGATATTTTTTATGTAGTTTTAGAATTTCACAGTTTGTCTATTCCTTGATGAAAGAAAAGTGATTTTTGTCCTTTTTGGTGAAAACGTTGAAATTACAGGATTTGTCTACCTTGAATGTTTACCCTTCTCTCCAACCAGTCAAATTCAGCTTAGAATTTTATTCTCAGCTTTTGGAGATTGTGTTCGCTTCATGTTACAATTCACAATATTTAACCTTCGGTCGCAATTTGTTGCGAACCAACAAACCTAAAAATTATGGAGCAAACTCGCTTGGATCAGCTTGATAAATTGTGTTTTGGGAGAGCAGCTTTTGCTGAACCGTCCAAACCTATGCTATAGGGCAAACTGATCTTCTCTAAAATTGTTCAGATTTTTTCTTTCTCTTTTTGACATGTTTAATGACAATCCTTAAAATTCGAGCGTTGATGAATTAAAACCCAACTAAGAATTCCAAAAGAAAGAATTATGATTCAGTGTAAATGTAATCACAAGATGGGACGTTGTGTGTCCAGTTTAAAGACCTTTAGCGAAGTTGTTAATTGAAAGGTTTTATTTTTTAACTCCCGGCAGGAAACTAATACAAAATGCAGATAGCCACCTAATTTTGCGCGGAGAAAATGTAATTTTACATTTGTATAATGCGAGACATTTAGTACGCCCGAGTCTTGAAAAAAAAAAATGGGGAAATGAACAGTTTAGACTACCGTCTCTGAGATAATTTGAAGTGCTTTGATTTGATATTATAGCCTTGGTTTGTTTTTATCATCTACATTCCCTATGGAGGAATCATTTGACTTATGACCGATTTTCATGAAATATTAACCATGTAAGTGAAAAACTCCAAATAACTTCTACTTTCAACACACTATTGCGAAAACTTCAAAACTAGTTTGGTCTACGACCTTGTCAGTTGATAACAAACAAAAATCTAAAAATTTTTCACTTTCAAATTTTTAGATTTTTGTCTGTTATCAACCAACGAGGTCGTAGACGAAACTAGTTTTGAAGTTTTCCCAGTAGTGTGTTGAAAGTAGAAGTTAGTTGGAGTTTTTCACTTACATTGTTAATATGACGTTAAGTAACACTTTCTTGGCTTCGACAAGTAGAATTTCACAACGATTTTCATGTAGCTTACCAGAATTCTCAATCTCGGTAATCATTTCGAAGAGGGCCTACCATTATTGCTGACAAAATTTCTGATAGTGTGTTGGAACAGGACGGCAGTGATTGACTCCTGGTTGCAATAATTCAAACCTTTTTCGATCCAAGATAAGAGCCTGAGCTAACATGGCCATTCGGGGTTCAAACGAACTTCCAACAAGTGATTTTGGTGAAGATTGAATTGGTTTTGTCGAAAAGTTTGTAACCCGACGAAATTGCAGGTGCAATCTGCTCCCGGTGAAATCGCGATTGAACTCTGAACGTGGACTGAACACCAATCACAAGTTTAGAATAGTTACATTAACTTAAAACAAAAGAGGCAGACAGGTTAGGAACTGAAGTATTCGCCAAATTTTATGAACTTTTTATTAATGGTAGTATTTAAGGTACAAATGAATTTTTTTTTTTCATATTTATGCTCTATTTTAGTATTTGGAACCGTAAAATCAGGGTCTCATATTAGAATCCGCAATTTCTTAATTGCTCCACTTCATTCTTTATGGTCAAAGGACGAAAGGAAGATAACATTTCTACAGAGGGCCTAGTTGAGCGAGTTTAGATCAACAATTTTTCTCGCGCGTAACTATTTTGAATAATTATTCTGAATAATGTTCATTTGAATTTCTTGATTTGGTTCGGAGCGCACTTTTTAGACCAGTCGAGTGTTCCCTGAAGGCATAACGTAAATATCCGCTTGAAAACTTGTTTGTACGTGACCGGTAGATCTTTGAAGAAGCAAAGTCTCCATCAAACAGATGTGTTCGTGTAGGTTTCCTAGAACAAACAACACGTGGGTTTGTGACACATCACTAAGTGTTCTTTTATGTACCAGCTGAAGTCAGAATTGGTACTTAAGCTACAGACCATGTCTGGAAATATCCGGCACCTTAAAACCGGCCATAAAGTGAAAAAAGGATTCTTTTGATTAAGGTTTGGAGAACTTTGCCGCGTAGTCTTAGAAATCCGCTTTTGTTGTCACAGTAACCCTGATAGAGAGCACACATAATGTCGAACAACTACTCATTTTGAACGCGCGCTGTTGGCGCTCATTTTTCGTTGTCATGTCTTTTACTGAATAATTTTCTATTCTGTAAATTTCTAATTGGTTGTAATGAACGCAACAAAAAAATTTGAACAGCCAAGCTCTTTCCCTCCCTTTCTTGTCATACAAGCAACACTTGTGAAACGATATGTGAAAGCAGAATCTATGTTATTGTGTTTTTGCTCAGTTATTAAAATCATGTCTTTTCTCTCCGTTTTCCCAATAGCCATGAGGTGAACAGCTGGGACAGGAATACGTTCAGGTGCATAAACGGAATCTAAGCAAAGGAGCTGTTTTCAGCGTGAATCGTCGACAATTTATATGAACGGTAAATCTACAGTCCAGCTGAGAAAATTTGCTAAGTCATGTGTGGATCGAAAGAGAGGTTCATGGTATCTCAAAAGGAACATTTTTGATGTGGGTTGATTTCCAAAAGGACAGGATTTTAATCACTGGTAGGAAAGATATAAGAGACAGTTTGGCTGTAGAGATAAACAAGCCAGTAGATTGATAAGTATTTCATTTAAAATCAATTTTGCAAAAAGCGCGCCATTTTGGCCCCATAGAGAAAAAGGAACCATTACTGTCATTAAAATGTAATAAAAACGCCAGGTTCACAATTAAAAAAAAAAAAAAAAAAATACATGTACATTTTTATATATATGTAAATGCAATTTTATTTGTTGCCGTCGGATGGTTTTCCTTCTCCTCTGGCGGCTTAAAGCAGTTGCAAGTTATCTCGATGTTTTTAATACGAATTCGAGGCTTTTTTCCAATTCATGAGTTTCCAAGCAAAGAGCATTGACATCTCATTCGAATATATAATTGTGCTGAGACTAAGAAAAACACTTCAAAACAACACATTTGACCGTAAAATTACAACGAAGAAATCAGGTGCATTTTATAAAAATTTCCAATTTGATGATAGCTCTGAATGAAAATTGCACGAGTTAATCATTTACATGCACCGATATAACGGCCAGAAAAGACATTTCATTTGGTGACATTTTGCGCTCTTACATGCATCAATACAAAGGGGAGAGAGGAAAATAAGAATTATTGTTTGAAACATAAAAACGTAAATCTTTGACATAAATTAGAAAATTTTTCGAGAAACTAAAAATCTACATGCATATGCAATTTCTTTCACGATTACATGAAATCTTTGATTAGTTATATAGTGTATAAATGAAGAAGTTCAACTTTTTAACAGTATCTGCAAACAGATTCAATTGCACTGAGTTCTTTTAACGATGCCGTTTGTTCTTTTGCGTTACGGACTAGGGAATGCAATTGATAATTGCAACCCTTTTAGTCATAATGGTAAACAAGAGATGTCAAATTGTTGATTCTAATCTCAAACGAGATATCCTTTAATGGCAAACGAGCCACATAAAGTTAATTACAAACAAGCGGTGTTCTGAATCATGTAGATGCAGTTGATATTTTTTTAAATAATTTTCATGAACCGACCTCTTGAATTGTTAGCTGGTTCCAAATGTTATTCAAATAGAAAAACCATCATATATTGTTTGGTGGAAAGTTTGCCTTCATGTCGATTGAGGGAGAAAAATTACTTGTTTTTAATCTGTTTGGTTTGAATGAGTAATGACGTACTATCTGTTGCCATTCATATTTAAGACTCATGTAAACTCAGCCTGAAATCCTGTATTTTCCTTGCCTTTTCATTAATTTCACCTTGATTTAACAATCGCACACGGTATGGTATTAAAAAGTGTTTCGTGACTGCTTTTTTTAATCGGGTGATGAATTTTCAAAATACATCTCAGTCAAAATGGTTGAGTTTCTCGAAACTGAAATTGGAAAATAAAAAAAATTATACTCACTATTGTCTTTTTGAAATCAGTTGCCTGTTCACCAAATTACCTCTCCTTTCAGATTTGATTACCTCAAATTTTCGAATCTTTTCTCCACCGCTCATTTAATGCGACTGAGGCGGATTTGAGTCTGTTTGAGTATGTCTGCAGGGACATTGGGACAAGGACGTAAGCCTTTTTGGTGTATTTACTAATTTCAAAACTTCGCGAAGCCCTGGGTCTGCCAATCAACAATTCTTATAAACTAAATCTCATAGATATCACCAGCTAAACTGGGGATATTAGAAGATATCACGTAATGATAAGTATAATTAAAAACTGGATCATTGGATAATGCAATTCAAGACTTTTTATTGGCTTAGCCATTATGGTATATGAGCCAATATAC

>SpiHIFβ cDNA

TGCATAGTGAATTTCCAGTCTGGGATTGTCTTCCTTTATCGAAAGACTCCTGCCACAGAGTATCATTTCGCTCCAGCTGTTATTTTGTGAAATTGTTACAGGTTTTTGTCGCATTCGGAGGTAATTTGGTCGGGCTGTTATCCAAAGGAGGCTTGCAGAAACTATTTGCTGGGATGCCGCCATGATACTAAGGTAGACTCTTTTCATGGTTGTCTGCCGATTCACTCGCTTGGAACTCCAACTTATTGCTAATGTGTAAGTGTAACTAACAGCAGCCAAAATGGATTCACTAGACGACTTCGACCCGTCCAAAATGGACGATTATAACGACGATGATGATGGAATACAGGACAAGTCTTCCCATGGTAGACTTCGAAAGAGAAAAGCCAATCAGTCCTTCATGATCAAAGAGGAAGACTCGGATGATGATGATCTCGAAAAGGGTGATCAAAAAATGCCCAGAACGTCGGACGAGTCTAACAAAGAGAAGTTTGCAAGGGAGAACCATAGTGAGATTGAGAGGCGAAGAAGGAATAAAATGAATGCTTATATAAACGAGTTATCTGATATGGTACCATCGTGTAATGGATTGGCTCGCAAACCAGATAAACTCACTGTGCTTCGAATGGCTGTGAACTATATGAAATCTCTCAGAGGACCAGGCCAAGGTCCTAGTGATGTATCATACAAGCCTTCATTTCTCTCAGATCAAGAACTTAAGCACCTTATATTAGAGGCAGCAGATGGTTTCTTGTTTGTTGTGAATTGCCGCACTGGAACTGTGGTGTATGTTTCAGACTCTATAACTCCAGTCCTTAACCAACCTCAGAGTGCTTGGATGAATCAGGAAATTTTTGACCTTGTCCATCCTGATGATACAGAGAAAATAAAAGACCAGTTATCTGCTACAGAGTCCCCTGATGCAGGAAGGGTCCTTGACTTGAAGACTGGAAGTGTGAAAAAGGACAGCCATGGAGCTCTGTCAAGAATGTATTCCAACTCAAGAAGAAACTTCATTTGTCGCATGAGATGTGGAAAAGAAGAGAGTGAGAATGTTGCAAGGGGGAAGGACAATCGTTTAAAGCTACAAGATGATGAATATGCCATTGTGCACTGCACTGGTTATATTAAGTCTTTTGGAGGCAGCACTGCTGTATGTGCACCAGGAGATGCAGATGCCTCAGAGAACAGCTGTTTGGTAACTATTGGAAAACTTCAGCCTACAAGTATGCCACAGAGTTCTGACTTGTTGGATTCCCCACCTGTGACAGAATTTATCTCTAGGCACAGCTTGGATGGCAAATTTACTTTTGTTGACCAGAGAGTTTCAGATGTGCTAGGGTACAAACCCCAAGAACTTTTGAATGAAGTGTGTTATGACTTTTTCCATCCTGATGACCTGGAACATATGATGGAAAGTTATCAACAAGTAATGAAACTGAAGGGACAAACACTTTCTGTGAGGTATAGATTCCGTGCCAAGGATGGCAGCTGGGTGTGGCTACGAACAAGCTGTCACAGTTTTCAGAATCCTTACACTGATGAAGCAGAATACATTGTTTGCACTAACTGCCGTGGTCAACCTGGCAGTATGCAGCCTGTCACCCTAAACCTGTCCAGCCCAACTATAAACTCCACTCGCAGTGGGCCACTGACCGGCAGTACAGATTATTCTGACATATCTCCTCGCACAGACCTAACAAGCCCTGACTCTGGAGTGTCCATGTATCAGAGACACGCCCAGCTTGCTCCAGTGCGAGATGATGCCATTCATCTTTATACACAAGGAGGTGCAGTCCCAAAAGCATCCCAACGTTTGGAAGGTGGCTTGGAAGCTCTTTCTAAGGCCAGTGAGCTTACTGACAAGAGAGGTAATGTTTCACCTAACACTCCGACTGCTCAAGTACCCAAGAAATCTGACGGCTCAATGGTTATTCCAGTAACTAGTGCAACAACACAGAGACTTCCACCAGGAATTATTCAACATGCCATGGAACATGGCACTGTTGACTTGGACAGTGCTGAGTTTTCTGCTGGCCAAGCTGGCAGTCTGCTTGCAGCCATGGTTCAGCGGAGAAATGCCATTGCTGCAGTTTCTCCAGGCCAAACACAGCAGGGTGGGTACCCTCTCATTTCCCAGATGAATGGCAACATGGCCACTATTATGAGACCTGGAAATATGGGAGGACAACAAAGAGCCATGGGTCCAATGTCACCAATGGAGATTATGAGAGCACAAATTCCACGCAACATGGCTGACATGAATTCCCCTGGAATGATGGTTTATGGCAGTGCCACAACAACTCGGAATGGTCAAACCCCTGGCAAGAACACAGTTGACACTGGCCAAAAGAGTGGGTATCCACAGGTAATGAATGATGGTGTAAGTGGAAGAGATATGATGTCTGGAGCATGGGCACACCAGAATGTTATGGTGAGACCTATTAGAGAAGGTGAGATGAAGCCAACAGCACCTGGAGGAATGCCTCTTGCATCACAGGTGGATGCTGCCACACAACAAGGGATGTATACTGGAATGAGACCACCTGGGGACCCATCCAACTTGCGAGGCCCTACACAGGGATATCATTACTACCAATAACCCTATAAAGCCCAGAAGTAGAAAATATACTCACTGAAACACATGAATCAGGACTTACTTTACTTTGTTTGTGCAGCAAAGTCAAATCTGCCTAAATGGATTTGCCACTGACATCTCTCATGGTTGGTGAAAATGCAGAAGGCTGAATGTATATGTATCACGGAAAAGATCGATAATTTTCACTGGTGTTATATAACCATTATGGTAATTAGCAAGAGGCAAAGATTATTTGCCAAAAAACTTTGAGAATGAAGTCATTACAGGGATTGCATCAATATGGAGACAGTGGGTATCACCAGACTTATCATATTTAACAAAGCTAACAAGCAACTATGATGTCCAAAAGATCTGCATAAGAATTTCCTAAAACATATTTCACAACTGTAGACCTGTTGAAACTGTAGAAGTGTGTAAGTAACTTGTCTTTTTTAAATAATAGGATTACCATAATTATGGAAGATTTTTATCTAGTCCCTTTATGCTCTCATCTTCATTTCACTGTTTATTATCTAACTTTCATTTTAGAGAGAACCAACAAGTGCTAGACAAATTATTTTTTTGGACCCATGGTATCTACCATTAATATTGTTTGTGATAAAAAAATAGTCTAGGTGTTTCGGCAACCTTGAAATGTACAAGAGCTATATACTTACTTCCATATGCATAAGCCTTTAAACTGTGCATTGTTGTGGGAACCCTCACACTAGCTTGGTTAGTTCTGTTTTACACAACATCTTCTCTTTTGATACCATAATATCCAACCTCATTCTTGCAGGAACTTTTTATCTTAAAGATCTGTCACTGTACTCTCTAACAGGATTATTTTCTACCAGCAAAACAATTAAACTGACATTAAATAGTTAATATAAATGATCTCCAAGCTAAAAGGAAACTTATATCATAGGCTTAACATGGGATTGACATGGACTGTTCACAAGAAAACCTTTGCATTCAATTGAAAGTGAACATTTTTGTATTGTCCGAGTACATATACTAAAGAGATTTTTATTGTTCATATCCTTTAAACATGACACTGTCCTCTGTGATCTTCCTGTGGTCTCTAGTTTACTTGTAGTAGCAGTGACTTTCACCTTGAGTGTTGTGGTTTTCATACCTCCAAAAATAACCACAAAGCAAGCTTACATAGCTGGAGGTATTGTTCTTTGTGTGTGAGGTCAAAAGACGTATCTCCTGCATTTTAATAAAGGTTCGTGTTAATTATATGCAATCTGTGTTTATCTCCACTCTCCTTAGCCATGGCATGATACTTCCCCTTCTTACCAAACTAACACTTTAGATTCATGTGTTATCTGCCCTAATCTCTTCAATCCTCATCCTGATTTGCTCTTTCTTGGTTAATTATTAATCCTTTTGCTTTTTCCTGTCCCACCAAACTTGCAGTATGCTTCAAGGTTTAGGGAATGGTTTCACTGCCACTAACCTTGCCTTGCACATCAGTAAGACTTCAGTGAAATTGGCTGCTATAGGAAATCTAACTGTTGTGCAAACAATCCTGCTAGCCATGTAATCTGTGACAAAAGGTCCATGCGAGTTACAGAAAAAGAGGTATCAAAGAGCTCCAGATAGTTTGTAGTATATTTGTGGACAAAAGTATTGGGTACATAATTACTGGTGCACAGAAAGACATTTGACAAAAAAAATTTTTTTTCAGAGAGACTTTGTTTTATTAAAAGTAGGTGTAGCCTTAGATACTGTTGTGGTAGTGTTGTAATTAATAATAAATCTTATTCTGAGGTGTCTTCTGTTTATTGTTGTAGTTTTATGTTTGTATCAGTAGGTCATTAAGTGGCAGAAAAAGAACTGTCATTACATTACGTCATTTTTGTTAGTCTCGATTAATTTTAATTTTTAGCAGTGATCCACAGGTCCAGACCCTCGCTACTACTTTCAGACCTGTAGGTTTTCTTTACCACCAGTCAGATTTGAGGTATTTTTGTCATCTTGTCTTGAATAAAGGGAAGGGGGGGGGGTTAGCATGGGTCTGTGGACCTTCCTATCAAAGGTTTGTTGGCAACCCCCTTTAGCTGTCTTTCATTAACCAATGAATTGCCCCAGAAGAGCTGGTCATGTTACTTAAATAACACCCTCTTGCAGATCACTTCCTACAATTTCCTCACCGCATTGTGGTACAAAATTGTGAAGAGAAGTTACATGTTAATTACATCTGGGAGTTTGCAAGTGAAGATTAAACTTAATTCACTGTTACCAAAGTAACAAATTAACGGTTTGTTTGTGGACTTCCCGAACCAACATTTTGGATTCATTTTCAATCTGTCATAATCTTGTCATTCCTCAGCCCCACTTGATCTTTCTTTGTTAATTGTTAATGGCATAAAATGAAAACTTTCTTGAAGTCATGGAATCCCAAATCATTTTACACCCACACATTCTTTAGCAGAGTTGGGATCTGCTACTTTCTTAAATGGCCTTTTTTTTTGTAGAAAGTTGATACATTGATAGTATACATACTACTAATTTTTCAAAGATTGGAGATGAGTGGTTATGGCCTTTCAGTCATTACGAAGTGACAACCTTGAAACTCTACAGAGGAGTGAGGGTGTAAGCACACAGGGCAACACTAGATATGTAATAGATAAGAAAAATATGAGTTTATTTAAGTGAATAAAGGCTGATAGTTTCG

>Genome scaffold containing the SpiHIFβ 

TCCTCGTGCTCAACCATTCTTGTGAAAATGAGTTTAATTTGCATGTGAATGGAAACTTATCATATGAAAGGATTAGCACGAGGACTCGTTTTGAAAATGAGGCCAAGGGTAATCCACAAATGGGCTATTGCTTAATCACCCACTCACGAAAATTCGCTCCTTTCCCCTTTGGCTACTTGCAACACTCACCTAGATTTAAATAGCTGCTGAGATAACCCTGGGGTTTGGTCGACAGATAGTCATTTTTTGAAAAGCACTCTACACACATCAGGTTACACACCAAGTTTTCAAAGTGCACTTTATTCTTATTTACACGACGCCTCTGGTAAAGCTTCTCTCTCTTCCTGTCTCCTTTCTACTTTAATTACACAGGCTCATACGGGATTGATTTAGATTTGACTATTTACAAGTGTCAAACTCTCCAAATTCTTCTCACAGATCACTTTTACTGAAAGCTAATAATACAAGAGATTTCCCAACAAATAGAAGATAAACAGAAGAATAAATCTAATATTGTGGGGAGTTTCAACAGAATTTCTCATTCCTTTTTCTCCTTTGGCATCAAGCCTAGGATTAGCAGCAATTGAAAAAATAGCAAAGAAATTTGAATAGATCACTGACAACTGAAAAAATTGTATTTATTTCCTAATCTTGTCCAGGATGGCCATGTTGGGGTGCCTTCGTGTGTAAAAGAAGATATAGCCTTTGCTTCCACTCACATGGCCTTCTTCTGTTTGTGTTACATTGCTGTCATTAAAATTATACCATAGTCCTAAGAAGGAAAATTTCCAGTCATGAGTTCACCACTGTCCAGGAACCATGACACACCACTCTTCCATAAACATAGTAAAAGAGTCATGATTTGTAATATCGCCTGTATGTTTTCCCCATCAGAAAGTTGATATAAAGAAGCACCTTAAGTATTGTTAATGCCTGGATATTTTCCGTTTCATTTGATGTTAATGCTTAAATTGCTCCAAAAGTATAAGGACGATAATGCAAGATTTTTGCTTTACGTTTCCACTGTATCTAACCAATACGAGAAATACCAAAATAACTTGATAGGTTACCATCTTAATGTCCTCTCAATCAATTAATACTAATTTCCTAGATACAAATCACACATTCTCTAAGGAGTCCAAAAGCTTACAGTTCTTTAAAATCTCCTGTAATTTTATTGATATGAATACTAGCTTTTGGCCACATGGTAGAGACACATGAAACTACCAAACCTATCTTACTTACCATCGTGAAAAGCAAACGTTGTGTAATGTCCCGCACTAACTCTGCAAACAAATAACAAGTAATTTTAAAAATATTCTTTTTGCTATAAAAGTGACACTCCTACAATTATAAATCACAGTTACACGTAAATTCTTACCCACTGCCATGATGAACAACAACAGCCGCAAGATCAAAAAGAAATGCCTTTGGGTTCTCATCCTGACGAGAGATACACATGTAATTAACCAGGGCAGAAAGAATTAAGCAACTGCACTTTTCCTCTGCAACAGCAATATGACACATTAACTAATAACAAAGATACACTATTCATCAAAGTCAAAAGGACTTCAATCAGTCAACAGAACTGTAAAAAGCTATCACAGGACTTCCACCAAAAAAATAAAATATCGATCTTGATTGTAAATACTGTAGTGATTCAATTTGGCACCCTTGTTCCAATAAGTACCCCCAATTAATTTACGAAAAAATGGTGGTCTTCATCTCAGTCTCTCAAGTATTATGTTCATGTGAGTTGTGCAGTTCTTTCAAGCAAATGATCTGGTCCTCAAATTTTATGCTGATTCCTTGGCTGGTTGTTCTAAGCCAGGAAGGATCCAGGGACAATAAGTCTGTTTTCCAGTTTGGTTGACCCTTTTCAAATAAACATTAAAACTAAATGTTTTGCACATTCTGCTGTTTCTGAAAAAGGAAAGAGGCACCTTGTCCCACAATAAGTGCCCTGTTTCAAATATGCACCCTTCTTTGAGGTACGAAAGTATATATTCTCCAGGCGCTAAATCCAATCATTATGGTATGCCGATCTCTTTAATCACCAAATAAAAGAAACTTGATGTTGTTCTAAAACTTTAAATGGCCATTCAACATCTCATAAGCGACATTTAAGACATTGCCAATACCCTTAGTTCAATATTACATTCACAAGGTTATTGGTTCTTACCTGGTCTTGTAAACAATAAGGACTCATGTCTAGTCCCCTCATGGGGAACTGTACATAAGTATCAACTTTGCTCCGATGAAACGCTTGAAATTTGAAACGTTTCAGATGCAAACACAACACCTACAAAACCATCAAGAATTTATCAGGCTAAGTTAAATTTGCAAAATATCAGAATAAAGCTGAAAAGAAAAGGCTTAAAAAAAAGACAAAAAGGATAAATATAAGAAACAGCAAGTTGTTGCTATTTGGTTCAAAACAGACAGCTTGACACAAAAATGTGACTAGAACAATTCTCTGAGAGGGAACAGTGATGATGAGATGCTAACGAGTTTCTATTATGGGTTCAGGAGTTTTCTGTGTTACTTCTACAGTTTCAATAACAACATTTATGTGTCAGCAATAAAAAAAGCAGAGTGAATGAGTTAGAACAAGGGAAATTTTCTCACATTTGGTAGTCTTTTAATCCAAAATTTCTTGGTAGACTTCTGTCTCTTTTTACAATTTGGACAAATGTATAGTTCAGACTCTGCTAGTTCTTCAAGTGATATAAAGTGATACAAGCAATCTGCAAACACAAATTGAGAAAATAACATATCATTCCAGAGAAAGTGTTTACTTTCTCCACAAAATAACACATCATCTGTGTGGAAAACAATGACATGGATTATAATAAGGATTTCTTTAAATACATCTTTACTAGATGTGAATGGAAGATTTGGCCAAAGGAAACAAAGCTTACTGCATCATTAACTGACATAATTACAGAGTCACATTATTGAATAAAATCTTGTATTCTGATTGGTTGATGAGGTGAGGTATTTACCGCGGAATTGAAAGTTGAAATTGGGGCAAAGTGATATTGTTTCACATCTATGTATACCTAACAAACTGTAAGTAATGCTTTCTTCTTTTTACGTTTTTATTCAATAATGCAATTCAAGAAAAATCCATTACTTGACTTGTGAATTCTATGTTAAATTGCACTTGAAAACCGATACCACACTCATTGCTTTGAGATTCGTGCAATATTGGTTTTAGTCAGCAATTTAAAGCCAAATTCCCTCATCAAGTAATGAATTTTCCTACAGAACCCATGAGGGTAGTCTTGTGGATATAACTTTAATGCTATAAAGTACTTGATCCTCACCTTGAAGTCGACAAACTTGAGGATCTCTAGTCTTTGATTTTCGTACTTGAAATTCTGCTGGAATTTCTAGTGACAAATCTGTTATTAACAAAGACAAAGACTTAGTAGGAATTCCTACAATCACATCTTGCAGGTATTTCATTAACCCTTACAGGGYGTAAAATTGCGCCTAATACAGGCGCCAATGCGACTAAATTTTTCACTTTGGCAACCAAATCCTGAAAATTAGTTGCCAAATTGGCGATTAGAATTTCTAATCAAACCTTAGCTAGAGATCTAGTGATTGTTCAAGATTCGATAAGATAAATCTGCAGCAAAGTTCCTCGTTAAGCTCGTTTCCAAAACGCAGCACATGCACGATGTCAAACGTAAATCTACTGTCACTAGACGCCATCTTGGATTTAGGTAACCTATAATGTTGTATATGATCTATTCTAGTGTCCTCTTTGTAGCTCGTGCGAATTTAGCGGGCTCATTTTTGATGTTTGTACGACTGTATCACTATGACACGATAAATCTAGTACAGTGACAGGGTAAAAATTCTTTTTTGTGTGTGTCATAATGGCCACCAACTTTTTTGGAATTGGCTACCACTTTAAAATATTTAGGAGCCAAGTGGCTACCGGAAAAAAACTTAATTTTACGCCCTGCCTTAACACCCTAACATCAGTATGCAAATTCTCCTCAACGCCCTCTACACATTTCCTTAGATGCTGAAAAGGAGATTTTATTTAATGATCAAGAGCTTCTTTAATTGGTGATCATTTCTTTTATTCTCCTGACCTTAAGATGTGATTCAGGGCTGATATGACAAGGTGAAATTTGATGCTTGTCCCTCTTGGGTGTCAAAAGGTAAATGCTAAATAAACTACTAACCTAGAAATGGATCATACTTCTTTGACTCCGTTCCACAAACTAAACATTTGACCTTAAAATAATAATGATAAAAAAATCGAAACATTAACATAAAATAAAAATGTTTCATAAGAAATCATTGTAATATCTCCATTTCTGGTGTACCTCGCTATGTAATAGACCACCAAAGATCCCTGTAATGATGGTTTCTGTTGTTCCAAAACTTTTTGGAGACAGAAGAAGTTCACTGTGTAATCTGTCCAACAGATAGTGCATGAATTCATGGGCATCTTGTTGTTGGTAGCCTCTGTGGTAAAAAAGGTGATTTGAACCACAATTTTCATTAACAAATGTTCTGGCAAGAAAATACCATACTTACTTAGAAAAAAAGCACCTCTCTTGGATAATTGCCACATTTTGGACAGAAAAATACTCCAATTCTCCAATGCATGAAAACACCTGGTACAAAATTCAGAGACCCCCATGATCTAAACCTTAATGAAACTGCTGGGTAGATTTTCTTTATCATCTCATCACTATCAAAGGTAACAAAGAGGTAACTTACCTGAATCTTGGGACTACTTTCCACACAACAGAAAACAGAGCATCAGGAGAGTGAGTGACTCCTCCACCCTGCCATAGTGCACACAGTACTTTTCGTAATTCTTCCACAATGGATCTGTACAGGTCAAGAGAAAGTAACAAATAAGATAAAATAAATTTCAATTTCAAAATAATTTTTTTGGACAATCACATAGCTGTAGCATACTGTTTTAAATTAGTCATTTGTCCGACTTTTTCAAACTAGCACCTGTTCAATTAATTTATGCAGACAGTTTTCCATCATTTCAGATTAAGACAATGAATACGAGAAAAAGGAAACAAAGATCAACATGGTTTGTGAAATTTTAAATCAAGAAACAATATTCAACCACAAAACTTGGAAGTATAAGATTTTTGTTGAGAAGATCAAATAATTAACTATTACCAATAAATAACCTTTTGATCATTTGACTCCCAAGATCTCTAAAGCAATTCTCTTTACTGTCTTCCATGCAATGCTCATAATTTTAGTACAGAGAATTTGGTATTGGATCAAGCAATACCAAATTGATATATTGATATTTTTCTTAGTTCTTATTTCTTGTCTGCTTGATAGTGTTGATATTGTATGGAAAAAGTATATCTCAGTCATTCTGACAAATGAAAGGGTTAAAAACAGCTTTCTTACCCTTCATCTGTTTTCCTGCTTCTGGTAAAGTAGGGCTGTTTTCCACACGAGTTATCTGTTCTGAGCTCAAATGCAGGAAGGTCCTTGAAATAACAGCTGAATGATTGAATGTTACTGCACCAACAAAACAACAAATGTAAAAGGATTAAAACTTTACCCCCTAACATCAACATATACATTAAACAAACTATTCTCAACATATTTCCTGAAGAAGAATTTGTTTGACAATAAAGAGCTTCTTACTGGTTGACTAAGGAATACTATTATAGGAGAAATAAAATGCTTGTCATTCCTGGGGGGCCAAGGGCTATGCAACATGTTCTAATTCTCAAACAATATATAACATCACCTATATCAAACATATATATTACACACCTTAGAGATTGCAACACTGCATTCATGAAACATGTGTTCCCAAGATTTCGAAGACCTGGTAGGTACTTCACCCTTCCTATATTTTCTTGCTGGAGATAACATAAAACAAAGTTGTTTTTGATCATGATAAGGTTTATGACATGGCTTATTGAAGTCACAAATCTGTCAATGGTGGTCAATGTCTTTCTATTTCACTTAATATCTTGTTTCTACGAAAAAATCATAATTTACCTCTTCTATTTTCATTTCTAAAATACCTTCCTTTAATTTCAGCTGCAAAATCATTTTTACAAGGTTGGGCATTAGATTTTAAGGGCAATGAAATTTTTCAGATGCTCCATCTATCAGTTTCCAAACATCTGAAAAACTATCTCAACCAGTTATGAAATAGGTTGTTGTAGACCTGTTACTATTCTGTGATTGTCTGATTGCTTCAAAGTTACAAGCACTGCAATTATTTTATTTTCCAAAGCTTATGTTGTTGTTGGTTTTTTTTTCACTTTTGCACTCTTCATTGTTTAACTTACATTTGCTTTCAAAGCTTTCTTTTGTCTTTCAGGTGAAGTTGTATCCTGCAGACAACGTTTCCTATTAAAAATTGAGTTTTGTTAATCATTAACATCCCATTTCAAAAGGGCACATATGTTAATCAAGGGCCACAGATTAAATTTAGCCTGAAAAAATCCAGCATCAGTCAAACCACACATGAAACAAATTAAGCTTGAAAATACACATCTCTCTTTTTAAGAAGAAACAAAGGGAAGACAAAAGATTACTGTTACAACTTGTGCTCTGTTTTAGAAGAGTGTGGGGTTATACAGATATCTTTTCCAAAACTGCCATCCACTACTCTTACTAAATTAACTGCGATTCTCTGACAAACATTACGTCACGTAAAAACTTGCAGCGCTGTGATTCATGGGCTTGAAAACAACATGGCGGCCGATGATAAAACGAATAACGCAATCTTCTAATGTAGTTTATAACCCTAACGACGTAATAGAAAGAAACAATCACGGCCCACAAATTGAACTGAAAACATCTTAATGTAAGTGCAACGAGTTTAATTTCAAATGAAAAGAAATGTTTTCGCGATTTCCCCTTATTTTTCAACCGTTTTCTTTACATAAACGTACATACATACCTATTTTCTCTCTCTAAAATTATTTCGCGTACTCTCTGAATATCTCCCGATTTTGTATCGTTGATTACGAATTCGTCACAAGCATAGCTATAGAAAAATGTCAAAATTTCACAAAGGGTAAGCAAAAACGTCGAGCAAGAAATCGGAAACGAAGCTCCAATGACATCACCGAATTTCCTAGCGAGGTACAAATCAATGTGAAGTACAATGTGCTCACCAAAACGCAGACAAACTTGGATCCAAACACACCGAATGGTGTTGATTGTCTTCGTGGTGAGCTTTAGCATGACCATTCACATACCTGGGTAAAAATGACTGCTTGATTAATAAAACTAATCTATGAGTGAATAACTGATATCAGATTCATGGGAGAGCTAAACAGTGCGGCGACATTCCTACGTCTCGACACCCCCCTGACACAACAACTGAAACAACACCGTGATATACTATCGGCGATGTACCTTCCGCAGTTGATGACACCACAACGCAGACATATCCACGGACTTTTTGTTGATCTACAACCTAGAACACAGAAATTAAGTCAGAATTCTGAGCTCTTTAGAAACACCGGTAAAGGTGAAACCAAAAGAGCAGAAAGCGAGAACAGGGTAACTAATCGACCCTCTTCGATTGGGAAACCGTACTTGAGCACGTCCACTGTGCCGGTTTTTCACGGCGTATCTTTTGAATAAGTTTTGAACCAACTTTCACGGCTTGCCCAACGTGTGTGCACTCCATATAACCCAAAAAAGGCAAAATTCTCACGAAAATCACAACGAAAGGGTCAAATTTTCTACGGATCCCTCCTCTCCCAGGCGCTAACGCGAGCCATTCGTCATGCGCCGTCGGAGCATTCTGCGCGGACTGAACGGTTACTAATTTTCCAGCCAATCAGAAGGAAGCTTTACTCGGGTGAAGTCAAATAACCGGCTATTCAATTTAACCAATCAAATTAATCGATGTATCTTGACAGCCTTTAAGTGACGTTAGAGAATAACCAATCAGAGAGCTTCTTGTATTGTCCCTCGGCTTTCAGCTGACACTAAAATACCTGTTACTTAATATCTTTTCGATCGATGTGCAAGCAGAAATCGAGTTATTACAGACTGCAGCTTCTTTACAAAGGTAAGTCCAATGTGTTTTACACGATTAGCTGTCTTATCATATTGTTCTTTCGTGTATTAACTCTGCACGATACGTTTTTACATCGACCAAATTGTAATGTTCTAATGCAAGATTTCCATGTCTGTAGGCTAGCACATAGTTCTATGGCAACTGCAGCGCAAGAAAATTATCCAACTCTGATCATTTATGGAGTTTGCTTCATGGTCTGCCTACATGTGGCAGCTCTTGTATCCTGTATTTCTGATAAAATAAATGGTACAGTGTATTTAAATGTAAGATGAATTTACGATGCAGTCAACATTCTAAAAATAGACTTCCTGATGGTGACTTAATGGTATGCTGGAGTTGTAAGTTGTACATAGAGAGATGAGTTGTGAAAACTTTCCTCTACAGTAGTTTAACTCACCAAAAACACCACTGAAAATTGTACACCAAGCCTTAATATATGTTCACTAGGGTAAACTCCAGCAGAAGTATCCTAGATACACATGCAGTAAATTAAAAACCTAATAATCTTCTGGGTTATTTAATTATGAAATGCAATAAGCTGTACAGACAGGGTAAAATTTTAAAAAAAAGGTTTATTTCCTTAATGTTGGACTCAGGGATTTTGGGTTTTCAAAGTATTTACCACTCCAGATAAAGAACAACAAATAAAAGGTCAAAAGGGCCATTAGTCAAGAGAGAGAACATACAGGAATGATGATTCTGCCGCTGGACTTTGGTATCAAGGAAGAAAGAGTGGTGTTTGGAAATCGTACAAGTGGCTACTCCTAAATTGGTTAATATATTTTTATATAGATATTTTTTTGTGCCTATGGTGCAACAAACTGAAGGATAAGTAGAGAAGATGAAGGCATGGGCTGTTCAGACCAGTGTGAAATGAGCCTCCACTTGGTGTTGGGCTGAGCACAATTGTTGAAGCTATGAATGAACTACTGGATAGTCAGGCACAGGCAACATTCCAGCACAGTCTGCCAGCTTGCTCTTCCTTATAAGCTTCCATGCTAGTGCTTTAAATAGTTCCTGTGGAAGCCTGCTGTGAGGTTATTCAGATGCCAAGAAATGCTACAATGTTGTTTTAGAAATCAGACATTTTAATGATCATATTTTCTTTAGTACCAACTCAGAATTTTCGTACACTCACTTACAAACCAAGTATTTAAATACTTTTAAATATATTTCAGAGATAGGAGACACACTTATTTTTGTCAACCAGACAACTTATCCTTTAAGAAAGGTTGGAAAAACTGAAAGTTTAACCATTTACATCACCCTTTAGCTCCTAGTCTTTCATTACTAAATCTTGAGCTCTCCACTGTCTACCATGCAATTTCAATGAAGATAGTAGTAGTTGAGTTTTTGTTTTTTTTCCTCATCACTAGTCTGCTTGGTATTGTAAAGAGAAATTCTGTCTTCATCCAAGCCTAAATAAACTCATAATTTGGATATATCAACAAAGTTGAAATGATAAATTGGCCACCATAACGATTACAAAAGCTGACGTTTCGAGCGTTAGCCCTTCATCGGAGTGATGACGAAGGGCTAATGCTCAAAACGTCAGCTTTTGTAATCGTTACAGTGGCCAACTTATCATTTCAACTTTGTTGATATATCCAAATTATTTCGTTACTCCCACTGACACAGCCCCAAACAGTTTCTTTCGAAACTATCAGCCTTTATTCACTTAAATAAACTCATATTTTTCTTATCTATTACATATCTAGTGTTGCCCTGTGTGCTTACACCCTCACTCCTCTGTAGAGTTTCAAGGTTGTCACTTCGTAATGACTGAAAGGCCATAACCACTCATCTCCAATCTTTGAAAAATTAGTAGTATGTATACTATCAATGTATCAACTTTCTACAAAAAAAAAGGCCATTTAAGAGAGTAGCAGATCCCAACTCTGCTAAAGAATGTGTGGGTGTAAAATGATTTGGGATTCCATGACTTCAAGAAAGTTTTCATTTTATGCCATTAACAATTAACAAAGAAAGATCAAGTGGGGCTGAGGAATGACAAGATTATGACAGATTGAAAATGAATCCAAAATGTTGGTTCGGGAAGTCCACAAACAAACCGTTAATTTGTTACTTTGGTAACAGTGAATTAAGTTTAATCTTCACTTGCAAACTCCCAGATGTAATTAACATGTAACTTCTCTTCACAATTTTGTACCACAATGCGGTGAGGAAATTGTAGGAAGTGATCTGCAAGAGGGTGTTATTTAAGTAACATGACCAGCTCTTCTGGGGCAATTCATTGGTTAATGAAAGACAGCTAAAGGGGGTTGCCAACAAACCTTTGATAGGAAGGTCCACAGACCCATGCTAACCCCCCCCCCTTCCCTTTATTCAAGACAAGATGACAAAAATACCTCAAATCTGACTGGTGGTAAAGAAAACCTACAGGTCTGAAAGTAGTAGCGAGGGTCTGGACCTGTGGATCACTGCTAAAAATTAAAATTAATCGAGACTAACAAAAATGACGTAATGTAATGACAGTTCTTTTTCTGCCACTTAATGACCTACTGATACAAACATAAAACTACAACAATAAACAGAAGACACCTCAGAATAAGATTTATTATTAATTACAACACTACCACAACAGTATCTAAGGCTACACCTACTTTTAATAAAACAAAGTCTCTCTGAAAAAAAATTTTTTTTGTCAAATGTCTTTCTGTGCACCAGTAATTATGTACCCAATACTTTTGTCCACAAATATACTACAAACTATCTGGAGCTCTTTGATACCTCTTTTTCTGTAACTCGCATGGACCTTTTGTCACAGATTACATGGCTAGCAGGATTGTTTGCACAACAGTTAGATTTCCTATAGCAGCCAATTTCACTGAAGTCTTACTGATGTGCAAGGCAAGGTTAGTGGCAGTGAAACCATTCCCTAAACCTTGAAGCATACTGCAAGTTTGGTGGGACAGGAAAAAGCAAAAGGATTAATAATTAACCAAGAAAGAGCAAATCAGGATGAGGATTGAAGAGATTAGGGCAGATAACACATGAATCTAAAGTGTTAGTTTGGTAAGAAGGGGAAGTATCATGCCATGGCTAAGGAGAGTGGAGATAAACACAGATTGCATATAATTAACACGAACCTTTATTAAAATGCAGGAGATACGTCTTTTGACCTCACACACAAAGAACAATACCTCCAGCTATGTAAGCTTGCTTTGTGGTTATTTTTGGAGGTATGAAAACCACAACACTCAAGGTGAAAGTCACTGCTACTACAAGTAAACTAGAGACCACAGGAAGATCACAGAGGACAGTGTCATGTTTAAAGGATATGAACAATAAAAATCTCTTTAGTATATGTACTCGGACAATACAAAAATGTTCACTTTCAATTGAATGCAAAGGTTTTCTTGTGAACAGTCCATGTCAATCCCATGTTAAGCCTATGATATAAGTTTCCTTTTAGCTTGGAGATCATTTATATTAACTATTTAATGTCAGTTTAATTGTTTTGCTGGTAGAAAATAATCCTGTTAGAGAGTACAGTGACAGATCTTTAAGATAAAAAGTTCCTGCAAGAATGAGGTTGGATATTATGGTATCAAAAGAGAAGATGTTGTGTAAAACAGAACTAACCAAGCTAGTGTGAGGGTTCCCACAACAATGCACAGTTTAAAGGCTTATGCATATGGAAGTAAGTATATAGCTCTTGTACATTTCAAGGTTGCCGAAACACCTAGACTATTTTTTTATCACAAACAATATTAATGGTAGATACCATGGGTCCAAAAAAATAATTTGTCTAGCACTTGTTGGTTCTCTCTAAAATGAAAGTTAGATAATAAACAGTGAAATGAAGATGAGAGCATAAAGGGACTAGATAAAAATCTTCCATAATTATGGTAATCCTATTATTTAAAAAAGACAAGTTACTTACACACTTCTACAGTTTCAACAGGTCTACAGTTGTGAAATATGTTTTAGGAAATTCTTATGCAGATCTTTTGGACATCATAGTTGCTTGTTAGCTTTGTTAAATATGATAAGTCTGGTGATACCCACTGTCTCCATATTGATGCAATCCCTGTAATGACTTCATTCTCAAAGTTTTTTGGCAAATAATCTTTGCCTCTTGCTAATTACCATAATGGTTATATAACACCAGTGAAAATTATCGATCTTTTCCGTGATACATATACATTCAGCCTTCTGCATTTTCACCAACCATGAGAGATGTCAGTGGCAAATCCATTTAGGCAGATTTGACTTTGCTGCACAAACAAAGTAAAGTAAGTCCTGATTCATGTGTTTCAGTGAGTATATTTTCTACTTCTGGGCTTTATAGGGTTATTGGTAGTAATGATATCCCTGTGTAGGGCCTCGCAAGTTGGATGGGTCCCCAGGTGGTCTCATTCCAGTATACATCCCTTGTTGTGTGGCAGCATCCACCTGTGATGCAAGAGGCATTCCTCCAGGTGCTGTTGGCTTCATCTCACCTTCTCTAATAGGTCTCACCATAACATTCTGGTGTGCCCATGCTCCAGACATCATATCTCTTCCACTTACACCATCATTCATTACCTGTGGATACCCACTCTTTTGGCCAGTGTCAACTGTGTTCTTGCCAGGGGTTTGACCATTCCGAGTTGTTGTGGCACTGCCATAAACCATCATTCCAGGGGAATTCATGTCAGCCATGTTGCGTGGAATTTGTGCTCTCATAATCTCCATTGGTGACATTGGACCCATGGCTCTTTGTTGTCCTCCCATATTTCCAGGTCTCATAATAGTGGCCATGTTGCCATTCATCTGGGAAATGAGAGGGTACCCACCCTGCTGTGTTTGGCCTGGAGAAACTGCAGCAATGGCATTTCTCCGCTGAACCATGGCTGCAAGCAGACTGCCAGCTTGGCCAGCAGAAAACTCAGCACTGTCCAAGTCAACTACAAAATAACACATGAATACAACAAAAGATTTACCAACTGTTCTTTGCTTCACTCTGAGCAGTGTCATAAATACACCTTCCAGGTAAAGGAGATTCCATTGATTACCTTATCACAAACTATCTCCAGTTCATCCCCCACCCCTTTAGAGACAAGAAAAAATGCTCCTCAATTCCTCTTTTGGGTGAATTATACCTGTATGCTAGCACAATGTTTTTTACTTCCTTTTGCAAAATCTTCCATATCAATTATTACTTTTGACATATTATAAAATCTGAGGAAAAAAACAACTGGTGGCAATGTACAATCAAACTAGATAACTTGTTTCTTTATTTTTTTTAACTGAGTTGATAATGTGAATTAGCCACTGTAAAGAGTTTATCAGTTGACATTTCAAGCATTAGCCCTTCCTCATTCTGTTGCATTACCATACTATTCCATTTAATCACATTCCTCAGAAAAAAAGTTAACAGTTACAACAAATAGGAGTAGCATACAAATTCAAAGTTCGGTGAAAATAATCTTTTTGGATGAAAAAATAATTTATTTATTCCTTTTAAGTGTCAATTCATTAAAATTGACTGTATTTGCCTATCTTGTTACAGTGCTGAAAATGAAATACTTGGATCAAACATTAACCCTCAAATAGACATCTAGTGTCTTACCAGTGCCATGTTCCATGGCATGTTGAATAATTCCTGGTGGAAGTCTCTGTGTTGTTGCACTAGTTACTGGAATAACCATTGAGCCGTCAGATTTCTTGGGTACTTGAGCAGTCGGAGTGTTAGGTGAAACATTACCTCTCTTGTCAGTAAGCTCACTGGCCTGGAAAGATAACCCTTCAATTAACTCTACTGGGTATATCATGATGTTGGCCAGAGAGAAGGAGAGAAGACAGTAAAAACATTTAAGTATTAAACAAGGATACTTCCTCACTCAGCTAGTAAGGCATCTCCTCATTTCTGTGGTATAATGTGACATTTACTGTTATGACCCCTGGATGAATGGTAAAAAGGTACATTACAAATTACCTGCTAGTAACATATAACTGATACTTCACTCTTGTAAAATAAGAGGAACCTGCAGCTTGCATAAGCCTCTTGGTTACTCTACAGGTTTCCTTGCCATATGCTAAATTCTCAGTTATATCATATTGATGTATTATGTAATTCCTGTTATTTCTTGTGATGTATTGTAATAACAAAATGGCAAAATAAACAAATACACCTTGTACCCACACTAAGCTACTGCTCACTGGTACCTATTCTTACTTCTGCATGGAAAGGTTCTCTGAGACAGTAAGACCCCAAACCTGACTAGAAGTCCAGGGAGAAATTTCAGTTGAAGATAAAGAAGCCTAAATTATGCTTAATGGCACAACCAGTGAGGATGTCAGCAAAATTCTAATTTCTACACCACCACAGTCTAGTAAGTGGTGTATTTTAGAGAGCAAAAATAAATTGGGGGTTACAAACCTTAGAAAGAGCTTCCAAGCCACCTTCCAAACGTTGGGATGCTGAAAAAAAAATTATTTCCATTCAAACATGAACTTTTTTATCTATATTTTACTGTCACATTGCCATAATATTATTTAAGAATGAATTAACTTAAGTGTGTCACCTTTTGGGACTGCACCTCCTTGTGTATAAAGATGAATGGCATCATCTCGCACTGGAGCAAGCTGGGCGTGTCTCTGATACATGGACACTCCAGAGTCAGGGCTTGTTAGGTCTGTGCGAGGAGATATGTCAGAATAATCTGTACTGCCGGTCAGTGGCCCACTGCGAGTGGAGTTTATAGTTGGGCTGGACAGGTTTAGGGTGACAGGCTGCATACTGCCACTACTCAAATAGCAGAAAAGAAGAAGTAGGTTAGTAAAGGTTACCATGATTAACATCCAGTCTTTGTACAACAAGAGTTTTCCCTATGATCTAATTCAAAGTACAAGTATGTAATCTAACTTGAGCTCCTATAATGTAAATGGATTATATGTACTCACGGTTGACCACGGCAGTTAGTGCAAACAATGTATTCTGCTTCATCAGTGTAAGGATTCTGAAAACTGTGACAGCTTGTTCGTAGCCACACCCAGCTGCCATCCTTGGCACGGAATCTATACCTCACAGAAAGTGTTTGTCCCTTCAGTTTCATTACTGCAATCATAAAATAATGCTAGTTGAGAAAAGGACTAGACTTGGTACAGGTTACATATGAAACCAATCAGTGATTTTGTTCCATTAATACAACCTTAAAATAAACTATCCAAAGAAGTTGTTTCCTAGTGTTTCTCTGCCATACAGTTTTTTTTTTTTTTTTTAAGACCAAACTTTATCAAAATGAAAACTAAATTTCTTGGTGATACACCAAACTCTTAATTCATTAATCTTGTACTGCATCCACAAATACCAAACAATGATTATTCCTAGTGACCCAAGGAAGCAATTTAAATCATATGTACATTACACTACCTCCAAGTTAGCTCAATTGATTAGAGCGCCGGACTGTTGAACGGGAGGTTGAGGGTTTGAGCCCCGGCAAAGTCTTAAAATACTGAGGAGGCTGTGCTACCTTTCTATAATATCAAGAAATGGATCTAGACTTTCTAGTCTTCTCATGTAAGGACGAATAAACCATAAGTCCAGTCTCCTGCATTTATTAATTAGTTAATTTGTTAGGCAGGGGACGTTAAAGAACCCACTCATGAGATTACTGTAATGTGGTCTGGCCTGTTTACTGGGAGTTGGGGGTAGGGGACCTTTATATGGATAACCTCATGGTCCACCTTTTCAAATATGTATCGTGTATTTGAATAAAGTAGTATTATTATATGTCAAGTTAATTTGAGGTAAATTATTTGTGAACATTCTACATTCTGGTTTTGTAACAGAAGTACCTTGTTGATAACTTTCCATCATATGTTCCAGGTCATCAGGATGGAAAAAGTCATAACACACTTCATTCAAAAGTTCTTGGGGTTTGTACCCTAGCACATCTGAAACTCTAGAAGACAAAACATTTAGCAATTTCTTGTTAAAAAAAATTTTACTTAAGAATTTTCAGAGATCAAATCTCTCTCACAAGTTCCTTACTGAAGAGAAAAAAAAATGCAAAAAAGTAAAACAGGAACACAAGTAAAGAAGCCATTGGAAATTTTTTTTTCACACACTCATTACTACAGTGTTCTAGTTACTTTACAATGTAACTGCCCTTCTCCCTTTAGTAGACCCACCAATGCACTTTAACATACAGTTGACTCTCAATAATTCAACTTAAACTCCTGTTAACTTGAAAGTTCAAATTATTGCAAGTCAAGTGTACTTTGAATTTTGATAAGATCAATGCGAGATTAATTTATTCTTGCACTTTCAATGTTTTAAACATTTTAGCATATATATATATTTAGGAACTGCTGTTATTAGGCATATTATGTTTTGACCGTGATTACTTGAATTTGAAATATTTCAAAGGAATTGTTCATTTGACTTATGATAGGTCAAGGTGTTTATTTTCCTTTTGTTATTCAAACCTTCCTTTTTTTTAAGAATTAGCATATATATATATACATTCTTGAACTTGATTTGAATGTATTTCAAAGAGCGCTCAACTCTTAGAGGAGCATTGATAATTATAACAAACTGGTTCAAGACTTCAGGGGTAGTTCAACGTTTTATTATGACAGCTCTGTTTTGTATGGTCGAAAATGACCACACCCATCAAGTAATTTCAACGATTGACCATTAACTTACAATGACTCAGGGTTTTCCTTTTCGGCCGGCGACCGGCGATTTTCGCCGCCTGCGAACGGCTCCGTCGCCGGCGACTAAAAGAGTCCTTGCTGTTATATAATTTAAAATATTTGAGATAAATAATAAACACTTTGTATGTTGTCTGACAATGACGTATAAGAAACGGAAATCGATAAAAAGTACATATTTCAGCAAAAAACGAAGTAGAATTTTTGCGCGTGTACTGGTANNNNNNNNNNNNNNNNNNNNNNNNNNNNNNNNNNNNNNNNNNNNNNNNNNNNNNNNNNNNNNNNNNNNNNTAAATTATTCAATTTTTGCATTTAAATAAATTACTATTTGAAAGGTCGATTATCTTCTTGATTTATCCGCTTTTAACTGTTTTAGTTAGAACAGATATACTTTAACTACGAACTACTTATTCTCACTAAGCCAAAGCATCACAAATATTATCTTGGGCCAAGTCCGAAGTAAAGTAAGTCTCAGGCGATCGTGTGGGATGCNNNNNNNNNNNNNNNNNNNNNNNNNNNNNNNNNNNNNNNNNNNNNNNNNNNNNNNNNNNNNNNNNNNNNNNNNNNNNNNNNNNNNNNNNNNNNNNNNNNNNNNNNNNNNNNNNNNNNNNNNNNNNNNNNNNNNNNNNNNNNNNNNNNNNNNNNNNNNNNNNNNNNNNNNNNNNNNNNNNNNNNNNNNNNNNNNNNNNNNNNNNNNNNNNNNNNNNNNNNNNNNNNNNNNNNNNNNNNNNNNNNNNNNNNNNNNNNNNNNNNNNNNNNNNNNNNNNNNNNNNNNNNNNNNNNNNNNNNNNNNNNNNNNNNNNNNNNNNNNNNNNNNNNNNNNNNNNNNNNNNNNNNNNNNNNNNNNNNNNNNNNNNNNNNNNNNNNNNNNNNNNNNNNNNNNNNNNNNNNNNNNNNNNNNNNNNNNNNNNNNNNNNNNNNNNNNNNNNNNNNNNNNNNNNNNNNNNNNNNNNNNNNNNNNNNNNNNNNNNNNNNNNNNNNNNNNNNNNNNNNNNNNNNNNNNNNNNNNNNNNNNNNNNNNNNNNNNNNNNNNNNNNNNNNNNNNNNNNNNNNNNNNNNNNNNNNNNNNNNNNNNNNNNNNNNNNNNNNNNNNNNNNNNNNNNNNNNNNNNNNNNNNNNNNNNNNNNNNNNNNNNNNNNNNNNNNNNNNNNNNNNNNNNNNNNNNNNNNNNNNNNNNNNNNNNNNNNNNNNNTGCTCAAACTAGAAGAGCCTAGTGAGCTATGCACAATGCTAGCGGCGATGACTCATCCCACAGTGCTCAATTTGGACACGAAAGCAGAGCTTTGTATGCCAAGGAGCTATATCTAACTGCAGACAGTACTGTTTCGGCCTTCTGGGCCTCATGTATATATATATGAGGCCCAGATTATATATAATTATAATCTGGGCCTATATATATAATTATATATATATAATTATATATAATATATATAAATATATATAAATATATATAATTATATATATATAATTATATATATATAATTATATATATATAATTATATATATTTATATATATTTATATATATTATATATAATTATATATATATAATTATATATATAGGCCCAGATTATAATTATATATAATCTGGGCCTCATATATATATACATGAGGCCCAGAAGGCCGAAACAGTACTGTCTGCAGTTAGATATAGCTCCTTGGCATACAAAGCTCTGCTTTCGTGTCCAAATTGAGCACTGTGGGATGAGTCATCGCCGCTAGCATTGTGCATAGCTCACTAGGCTCTTCTAGTTTGAGCACTCTGGCATGGCTTAGACAATACCACAAGTGTGGGATCGTTTGGGGATCCATATTGGTTCTCCCCCATCCTAGCATCAACACTGCACTGATGAGGCCCAGAAGGCGGAAACAGTACTGTCTGCAGTTAGTTATATATACATGTATTTTATTTTTCTCTTTTTGTTGTACCTGATTATTTGGATGTGACCGGAATAAAAAGAAGCAGAAGTTTGTTTTAATCATGTAAAAACTTTAGATGTACCCCTACCTCTGGTCAACAAAAGTAAATTTGCCATCCAAGCTGTGCCTAGAGATAAATTCTGTCACAGGTGGGGAATCCAACAAGTCAGAACTCTGTGGCATACTTGTAGGCTGAAGTTTTCCAATAGTTACCAAACAGCTGTTCTCTGAGGCATCTGCATCTCCTGGTGCACATACAGCAGTGCTGCCTCCAAAAGACTGTTGTGATGGAACATAATATATTATCTTCCTGTTTGACATATAGCCTTTGAGGAAATAGGAAGGAGAGGAAAGGGGNNNNNNNNNNNNNNNNNNNNNNNNNNNNNNNNNNNNNNNNNNNNNNNNNNNNNNNNNNNNNNNNNNNNNNNNNNNNNNNNNNNNNNNNNNNNNNNNNNNNNNNNNNNNNNNNNNNNNNNNNNNNNNNNNNNNNNNNNNNNNNNNNNNNNNNNNNNNNNNNNNNNNNNNNNNNNNNNNNNNNNNNNNNNNNNNNNNNNNNNNNNNNNNNNNNNNNNNNNNNNNNNNNNNNNNNNNNNNNNNNNGCACGCACTTCTACTTTTATTATCACCTAACAATCACATATTATGGTCACAAGAATAAAGAACTTGATCACCAACCAAAAAAAGCCCTTGATTGTTAACCAAATTCTCTGCATCAGCACCTTAGAGAACAGTATGGAGAATATGCATACTGATCTGAGGGTGTCAGGGGTCAACTACTAAAATCAGGCAAGAAGTTTAAGCAATTAGGAGGATATAAAAGTTCATTTGAAGATGTGAATCTTTACATTCTCATTTTAATTCATGGCCCCATACCTTAATATAACCAGTGCAGTGCACAATGGCATATTCATCATCTTGTAGCTTTAAACGATTGTCCTTCCCCCTTGCAACATTCTCACTCTCTTCTTTTCCACATCTCATGCGACAAATGAAGTTTCTTCTTGAGTTGGAATACATTCTTGACAGAGCTAAACAAAGCAAAGGGAAACAGAAATTGGTTTTTGTCAACAACACTTGAAGGGACAGAAAATTGAGTTATATCCATTTTCAACACTACTACAAAAAGTCTCCCTACCTCCATGGCTGTCCTTTTTCACACTTCCAGCTATACGGAAGATGAAAAAAATGAAGGCAAAAAAATCATTAACAATAAATAGTGTAGAAATAAAAAGTATCAAATCAATGCAACAAAGATTTGGAAATTTGAAGACCACTCGCAAAGATGTCACCAACATCATCTGACTTTTACCAAAGAACTACTTTGATAATGGCATTGTATTAACCTAAGGACTTGATCTGCTATACATTACTAAGGACTTGATCTGCTATACATTACTAAGGACTTGATCTGCTATACATTACTTACTCTTCAAGTCAAGGACCCTTCCTGCATCAGGGGACTCTGTAGCAGATAACTGGTCTTTTATTTTCTCTGTATCATCAGGATGGACAAGGTCAAAAATTTCCTGATTCATCCAAGCACTCTGTAAAACACAACAAACAAATAATAAAACAAACCTGAAAAATATCAAGTTTACCCCTGAAAAAAAAATAGGTAAAATAAAACACTCTTGGATGCCAAGGGGCCATTTGCAAACTGGGGAGGAGACTTTGCAGGAAATGCTGTTAAATCTCCACAGTTTGTTAGGTTACTAATTTACATCCCTGGTCAACAAGGGACAAGTTGTGAAAATTAAATGTTTTGCCAAAAAACACAACCTAGTGACCATTTTGCCATTTGCCAAAAACACAACCGAATGACCCAACAAGGCCTTGAACCTGCACCAGTTGACCAAGAGTCTGTTGTGCTAACATCTGGTCACTAACAATGAAATTACAGGGAAAAAAAACATGGTTCCATTCCCTTCATAAATGATACCACATTCAATTGAGCTTTTGTTGTTTAGTCTATTATTGTTTACAATGTTCACCTAATGAGTTGCTATTTGTTTATATAACTGACAACAAAATTTGGAGATTACCTGAGGTTGGTTAAGGACTGGAGTTATAGAGTCTGAAACATACACCACAGTTCCAGTGCGGCAATTCACAACAAACAAGAAACCATCTGCTGCCTGTGAACATGAACAAGTTTGCAAGTTTGTCTTTTAATACTTTAGCCTTAAACATGTCTCCATCTTCAAGAAGATGCAAGTACAATGTAATTATGCTTCCTCCTTATAACACACATGCTAAATTTAACAATGAAAAACTTTTGTATTTTGAATCAAAAACTTAAGCCCTACCTCTAATATAAGGTGCTTAAGTTCTTGATCTGAGAGAAATGAAGGCTTGTATGATACATCACTAGGACCTTGGCCTTTAGACAAGAAAAAAAAAAAACACTTTATTAAATATGATCAGAAGACAACTAAAACCTCTAGACAAGAAAAAAAAACAGTTTATTTAATATGATCAGAAGACAACTAAAACAGTCTTTCAAAACAGTCTTTAAAATAAAACTAAAAAAACTAGCAGCAAGATCAAACCTAGTAGTAAATGTCAACATGTACAGTGGAGACCAAGAATTAAAGTTGTCATGATGTGATTGGCATTGCTTCTTTAACTTCCAGTTTATCTCAACCCATAAGAATAGGTAACTTTTCACATTTTGTTCCAACTTGGAAATGAACATCACTGTATCCAATAAATTGTCATACACAGGATTATTCTCTCCGCATGAAACAGAAACCAATCAAGGCACACTCTTGGTTTTTTAATATTACTCACAGACTGAAAACACACATGATTTGTTGTAACATACCTGGTCCTCTGAGAGATTTCATATAGTTCACAGCCATTCGAAGCACAGTGAGTTTATCTGGTTTGCGAGCCAATCCATTACACGATGGTACCATATCAGATAACTCGTTTATATAAGCATTCATTTTATTCCTTCTTCGCCTCTCAATCTCACTATGGTTCTCCCTGTACAAAGAAAGAGTTATAGGGAGATTACTGGCCAGGTAACAAAGGCAAAAGCTACAACATGACATAAAGTACAGCTCTTAATACATGCACACAAGTCAAACTACATATATAATCAAAAGGAAGAGGGGATTGAAAGTTTCGGTCGTAAACTGAAGGGGGGTGGTGAGATGCAATCTAGCTCTTTGACCAGCAGCTTCCATTTAAAATTTACTTTGACCACATCAGACCAAGGGTGAGGCTAATGAAAACTTAAAATTAATTGGGGACAATTTGACTCCTAGTTACATTAGTGAGTTGAGCACAAAATGGTGTGCATTCCCAAGATACTATACAAGTTTTATCTCACTGATGTGGCTAAATTTTTTAAGAGGAAAGAAATTGGTAGGTTTGTCTGTTTTCTGAATGATGATCAATCAGCTGAAAGTGATTCTGTATACTACATTCCTTTCACACAGACCAAAAAAAAATTATACATAAAAAAAATCACAAGTTTATGTTTAGTTAGTATGACAGTGCTCAAGAATATTGGGTAATAGTTTTACTTAAATTTACCCTCATATATTTACCAGGCAAACAAATTGCCAACCAGATTGTTTCACAAGTACATTCTATGCGAAAGTATAAGGAACTCTCGATTACAACACCAATAAGTTCTTAAAAGTAGTTTAAGTGTAGTTTCGTCAAGTTCTATTTTTTAACAGAGACTGTTCTATGATAAGGAAAATTGCATTGCAAATGTCTTGACAAAAAAATTGAAAACATCTGCGAGAGGAAACGGAATAAAAGAACATCTTACCACCGGCTTTTAAAATTACAGCTAACAATGCAAACGTTTATGGTAAAAGTGTGTTCGATTGCCATAAATGATATATACATATATATAAATATGTATATAAGGAAAAGAGAATTACGAGTCGTTTACAATAAAATGGTTTTGGTAGCTTCGAAAATGTTTGCTGAAATAAAAACGTGGATGGTAGTTACCAAAATCATGTACGTGACTAAAAAAAAGAAACTAAATACCTTGCAAACTTCTCTTTGTTAGACTCGTCCGACGTTCTGTAAACATTAAAAGAAGGTTTATTAAAATAAAACACTATTACGCATGTTGCACTTGAAGCTTGAGGAAATATCTTTGCCTAGAGATCGGGCGTAACTTTCGTTACAAACAAGAGATTTGCTACTAAACAAACGACATGTTCTGTAACGTTGAAATCACGACCGCTGCCCGACTTTGGTCAAAATAATATAGACTACATTAACCAAAAGTGCAAGTTTCGATCGAAAGGCAAACAATCATAGGAAATAGATAAACTTGAGTAATTCAATGTCAAAACAAGCATTCGCCCGCTGAGTCCTTTGTTTTCAACGTCCCTCCTGCCGAACTTACGACCTAATTATTCTGAATTATCACAACTCTTTCACTGCACATCAAAGTTCAGACACTTCCTGTGGAGAAAATGGTCAATTTTTCATTACCTGGGCATTTTTTGATCACCCTTTTCGAGATCATCATCATCCGAGTCTTCCCTGTACGGTGCAAAAAGTAGGAATGTTTGAGAAGACGTTGAAATAACGCACACCACAACCAACCCTGGCCAGACATAGAATACGTCATAACTCAATACACGTTTCGTTCGAAGCCCACAAGTAAAACTCACTCTTTGATCATGAAGGACTGATTGGCTTTTCTCTTTCGAAGTCTACCATGGGAAGACTTGTCCTGTATTCCATCATCTGAGCAACAAGCACACAAATTAATTGCCTTCTGCATAAGAATCAACGCCATAAGCCTGCTTGTGTATGCAGGCATCACTACAAATTTGTTAGCAGTTTACATATTTTGCAGAATCATCTCTCTCAGATTTACTCACCATCGTCGTTATAATCGTCCATTTTGGACGGGTCGAAGTCGTCTAGTGAATCCATTTTGGCTGCTGTTAGTTACACTTACACATTAGCAATAAGTTGGAGTTCCAAGCGAGTGAATCGGCAGACAACCATGAAAAGAGTCTACCTTAGTATCATGGCGGCATCCCAGCAAATAGTTTCTGCAAGCCTCCTTTGGATAACAGCCCGACCAAATTACCTCCGAATGCGACAAAAACCTGTAACAATTTCACAAAATAACAGCTGGAGCGAAATGATACTCTGTGGCAGGAGTCTTTCGATAAAGGAAGACAATCCCAGACTGGAAATTCACTATGCAACGCACTAACGACCAGACAATCAATTCAGTACTTAGCCAGACTCAAGCGCAGCTTCTTCGATATGCAGCCAATCACAAAGTCTTTTAGATGTGTTTTGTTCTTTCTGACACCTAATTGCCCTAAATAACCAATGGTAAGACGTGTTAGAATTGACACCGGTGAGGAAGAAAACATAAAGCCATTCGAAAATGATAATAACAAGGCCTTAATCATGAGTGCGTCAACTAGCCAACTAAAGCAGCTCTTATAATAAGAATTATAGAAACTGTTTCAACAGCTGTCTTCTTTGCTCTATCAAGCGTAAACAACACAGATTCGACTTTTTCTGTTTGGAAAGGAAGCGTTTTGTTTACCAAAATAATTTACGAGAGTTAGGCAAGATCTATGATAAAATAACAGAGATGCTTTGCATATCAGTAAATAAAAAATTAGCCGAGTGTGCTGCAAGAACAACTCAGTAAAGAACTATATTTTATAATGTGACCCCACTACCCGTTCGAAGTCACGCCTGCGTAGTTAGCGTAGAAGACTGGGTTCTGACAAGACGTGACTCTTAACACAACAGAAGCAGCTAGCGGCACTAATTTTTCGAATAAATTTCACATGTGACCCTGCTACCCGTTCAAGGGTCACGCCTGCGTAGCTAGCGTAGGAGACTGGGTTCTTACAAGACGTGACTCTTTACACGGTGGTTCGTGGAAATGCGTCACAGCTACCCAACAGAAGCAGCAAGCGGCACTAGTTTTTCTACTAAATTTCAAATGTAATGCCCTCAGTGTTTCGAATGTCGAGACAATAGATTCGGAACTAGTTAGTATTAGAGCTTTCACATCAGAAAGCAGAATAGCACACGACAGAAGGGCTAGCTCTCCAAACGTCACGCCTAGGAGCTTCAAGTTGACAACTTAAACAAAGGCCAATCATGACATGGTCAGCAGTTGATAAAAACGAATTATCTTGCGATACCCTCCACAGACGCAGCACCACAGTTTGTTAGGAAACTTACCCCCTTTATTCATCTGATGTAAGAAGAGGGCGAGTAAATTATCTACCAACTTTGATTCGTTTAATACCGTCCGTCTATTATGATCCGAAAAAGCCAATAATGCGATCACAATAACAGATAAATTAGGGGATAAAGGACCGCCCTCACTTTTTAGTGGAGGGAAATCTAGGGAGATGGGGAGAGGAAAAGCTTCGCTGTGTATGTTGTAATTTAAGCAGGTTTCGGCACCGCATTTTCTTCGCGTAGAAAACTATTTCGTATTTTGCGGGCTGAAATGCTTCAACGTGATTGTCACGTTTCTAGGAGAAATTATGCGCCAATCACAATTCGACAAAACTCAAGGAACCATTAAACACGTGTACATTATAAACCAATCACAGCCCGCCAACGCAGTTCATTGAGAGTTGTCAAATTCAACGTGTATATTGCACCTCACGTTACAGCTGTTTATTTCTTCCATGTTCCGTGTTGCTTCGTTTCAAAATTCTCCGCCATAATGTTGATTTCCACAATTTTAATCTCTCTATTTGCATCGGTGGCTCTCTGTTCAGATGTCATTGAGCTCACCGATAGCAGTTTTGCATCCGGTGTAAAAGACAAGGATATTATGCTTGTAGAATTCTTCGCCCCGTGGTAAGAATTTGTACAGTTGTTGATCATCATTAGATTGATACGCACAGCTTGCGAACATTTTAATTTTTCTTCATAAACAGGTGTGGACACTGTAAGAAACTCGCTCCCGAGTACGAGACTGCGGCTACAAAACTAAAGAAAGAAGACCCGCCAATACCACTTGCCAAGGTACAGCTTCATCGTCAATTTCACTCTTCCGTTACTTCGTTTCCTACCCACTGTAGATCCAAATCAATCTCGCGTATTGATTTTATTGAATGAATTTAAACGTGCGGGTAATATAACTATTGTCTGTTTTGTTTGTTGATGACTTATTTGTTCCGTGCAGCTGAATATGATTTTAATGGTCTTGAAGCACCAGAATCCCTTGAGATTAAGACCCAAACAGCTGAAAATTGTTGATGCGCGCCGTTTAGTTGAAATTCTCTGCACTATGATAATGAGAGAAATGGAATATTCCCAATTTATGCTTGTTAGATGTGGTCAAACTGTCGAAGTATCTTGGAAAAGTAAAAAAATAATCGCACGTTTACATTCAGGTTTTGTGTAATTAATTATTGAGTACATCACCCTGGTTATATATAATACATTCTCACGGTTCGAATCTCTGGTTTTAAACGAAATTGTATGTGCCAGTTTCTACACCTGACTTGCTTTTGGGATATGTAAATGCAGGCAAGATAATTAAGACATTGAGATACCATTTTCTAACCTCCAAAAAAAAAAATAAAATAAAGGAAGTATGAGTGCACTGCAAATTAGTCTAAGTTGACTCAACTGTTTGACAGTGAAGATGAAGCTGGATTAATTATTCAACCTAATTGTTTGAGTATATCAACAAAGTTGAAATGGTAAATTGGCCACCGGTACAATTATAAAAGCTGATGTTTCGAGCGTTAGCCCTTCATCAGAGTGAATAGAGGAATTGTGGGTTGTGTGTGCTTTTAAAGGCAGAAAATGGAGCTACGTGATTGGTGGGAACATGGCAACGAGAAAAACACGAATAAATTAATGGAATGATGTGCACCTCTGCCAATGTAATCTATTGCATAACTTGCACTTATTGCAAAAAGATTTACATCGGCGAAACAGGCAGAAGAGTAGGTGACCGATTCCAAGAACACCTTCGCAATGTGGAGAGGAATGACAAAGACACATTGAAACCATTCGCTAGACATTTCAAGTTACCTAATCATTCTAAGCAACATATGGCAATCTGCGGTCTTTCCTTACACTTAGGTAGCATGGAAAGCCGTAAAACTNNNNNNNNNNNNNNNNNNNNNNNNNNNNNNNNNNNNNNNNNNNNNNNNNNGGCAATCTGCGGTCTTTCCTTACACTTAGGTAGCATGGAAAGCCGTAAAACTCTTGAACAAAAGCTCATCTTTCAAACTGGCAGTCTAAATCCCACAGGTACCAACCAACGCTTTTCATTCCACTAATTTATTCGTGTTTTTCTCGTTGCCATGTTCCCATCAATTACTTAGCTCTATTTTCTGCCTTTAAAAGCACACACAACCCACAATTCCTCTATTCGCTCCAACGAAGGGCTAACGCTCGAAATGTCAGCTTTTGTAATCGTTACGGTGGCCAATTTACCACTTCAACTTTGTTGATATATATATCCAAATTATTTCGTTACCCCCACCGACGCAGCCCCAAACAGTTTCTTTAGAAACTATCAGCCCTAATTCTTTGAGCATGATTTTTAAGTGATACTTATATGATTTAAAGAGGAGCTGTATTTTTATTTAGAATTTAAGCTTGGTGTTATAACCACCTTTTGAAGGTTGATGACAGCAAAATACACCTGCTTTATTAACCAAGTGTGAGGTCAAAATGGCTGGATATTGGCCAAGTTCTTTTTTTGTATTTTTATGGGCTGAGACAAAGTTGAGGTCCATAAAAATGCAAAAAAAAGATTGAGGTTAATATCCAGCCATCTTGACTGCATAAGCTTGGTTAATAAAGGATTTATTATATTGCCAAAAGATTTTGCTTTATTAAGAATTAAGAATGACTTGTTTACTTTGAGAGTTAGGAAAGAAAGCCAATTGTGTTGTAGCACAACAAACTCACTAGAGTTGTTTCTGTTTTCTTTGTTTTGACTATCTTCTGTTGCTTTTTGCATTTCCATCACCGAAATTGTCCAAGAATTAGGAAGATAGTTCTAATTCTAGCTTTTTCATGTGCAGGATCAATACGGGCAATCTTGAGTGGGCAAGATGGGCCCATCTTGCCCACTTGGGTAGCCAATCAGAACATAGGATTCACTTCACCTTTCCCACATGTGCTATGAGTGCTGTAATAATAGATAACTATCAACCAATCAGAGTGCAGATTATGATCATCCGCTGAAGATTAATCATACTAAAATTGTCAAACAGCTGTAGTGATGTTACTTTCCTTGGCAGTCATTTTTGGATTTGTCGCGCAAAACTCTCCTCCATTGGTTGGGAAAGATTGTTAGAGTGAAGTGACCACTGAATCACTGCAAAGGAGACTTGTGGTGACACTCAGCTTTATTGTCCAATCACACTCAATAGAGTGCAAATAATGCCTGAGATGAGTAAAAATATCACTCGGTATTTACACTCTAGAGAACTGGTTAGACAGGTTTTCCTGCATCATGCAAGCGATGAATTGGATGGAAGTTACAAAAAATGGGGGACAATTTAAATAATTTTTATGACGACAAAGAGTCGGAATGGCTGGCGAATATTGATCCAAGTTTATTAGATAAATGGAATAAAGAGAAGCAGTTAGGCAAAAATAAAATTTCATTATTTACTGAAGAAAATAGGAACAGCAACACGACAATGCACTAATTCCATTCTGCTTTGCGGTATACTTTTGTCGCACATTCCCTGTAACTAAATATCCACAAAATTCGTCCCCACTGAAATAGTAATTTAGCGTGTTGGATATTAAATAACTTATTAGATGTTTGGTGCGTCATATCGCTGATACGTATTGTAATGCAGCACAACTTGTAAAAATACTCAGCAATACTACACAACAAAACATCTAATAACTCCAGTCAAGGAGGTAATATAGCATGGGTGTGGAACAAGAGGTCACATGACCTTATAGCTGTTACATTATTGGATGAATGCTGCAATTTTCTTTTTAATTTACACCTGTTATAGGTTGACTGCACTGAGGCAGGGAAAGATTCTTGTAGTAAATATGGTGTCAGTGGTTATCCTACACTCAAGATTTTTCGCAATGGAGAAATGTCAAAGGATTATGATGGACCTCGTGATTCTGGTAAGTTTTGATAAATATAAAAGTTAATTAACTCAATGGATAGAACGCTGGACTGTTGTGTGGGAGGTCGAGGGTTTGAGCCCCGGCCAGACCAACACTCGAGGTCTTAAAATAATTGAGAAGAATATGCTGCCTTTCTATAATATCAAGAAATGGTTAGATATTCTAGTCTTCTTGGATAGGGATGAATAAATGGAGGTCCCGTCTCCTGCAGGGTTAGGGGTAGGAGANNNNNNNNNNNNNNNNNNNNNNNNNNNNNNNNNNNNNNNNNNNNNNNNNNNNNNNNNNNNNNNNNNNNNNNNNNNNNNNNNNNNNNNNNNNNNNNNNNNNNNNNNNGGATGTCCTCCCCTGGGGTGGGATGGGTGGGTTACTGGGCCCGCAGTGATTGGCACAAAGCTGTTGCGGTTTCCCTGCCTGTCGGCAAATCTTGAAAATAAATAAATAGTGATGATATTAATTTTTGGCTTTTAAAGAAATTGTCTTTTTTTTTTAAAAATATTTTTGGGATAAGAGTGACTTTAAAGCAAGTATGTATTCCTCTCAGGTAAAAAGACTGCCATAAACACTAGCAGATGAGCAGCACCCCCAGTTTGGCAACTCATTTTGGAAAAAAAGCTCTAGAAAAGAAATCACAATTAGAGTTTTGGGAGAGTTGTTTTATATTGGTTACTTCATTGAACATACATCAGGATGCGCTTAAAACAACTTTGAAAACTGAGAATACTTTGAATTCATACCTATAATTTTTAGCTGCAATGGCTTAGAATCACCTTTACTGACTTAGACTTCCATTTGATCTAAAAATTACGTGGAATTGTAAGGTAATTATTAAGCAAGAACACATGTTGCTGGTATAATTGTCATATAATAGAATTACCATTCAGGACCAGAACAATTTGGAATTTATTTATTAGGGATACCTGCTTCATGAAACCTGACTACGACCTACTTGCTAGAAGGAGGTGTTTGTTTTTTCCACGACACTGCAACTGAATCAACATTTTGCAAGAGCATTAGTCATTTCTTTGACTTTTTTTTTTTTATGCAAGTCTATTTTAACCCCAATTGTAGATATATTTTGAACCCGTCAGTTTCTTTTTTGCAGCGGGTATTATTAAATACATGAAGAAGCAGGCAGCACCCTCCTCCCAAGGTTAGTATTTAATTTAACATAATTTATTACACGACTCGGGCAGTCAGAGGGCCAATAACTAAAATCAACCAATCAAAATGCTCTATTGCCGGTCCAATATTCTCTAAGTATTGGACCGGGCATTGTCCCGAATGGCCCCAAATGATACCGCACTTGTGTCCCTTTGAACTTCAGTCGTATAATAAATCTCTTATTAACCAAGCTTGCTCGGGACCGTACTGGAAGAATATCGGCTCTCGGTCTTTTTTGTACAGACCTCGCTGCGCTCGGTCCATACCGTCAAGACCTCGGACCAATATTCTCCCAGTACGGCCCTCGCGCTTGGTTAATAAGATATATTTATATAGCGCTAACTCCACTAATTGACCAAAGCACTTTACAATTCATAATATTAAAATAATTGTTTTATTAAAAACGTTAAATTAAATTATTCAATTTTATTAAATTTTTAGCTCCCTTTTAATTTTAAGCTGCCACGTACGCTTACCATATTTGTAGATTATGGTATATTGGCTGATATACCATAATGGCTAAGCCAATAAAAAGTCTTGAATTGCATTATCCAATGATCTAGTTTTTAATAATTAAAAATATCCTACTGGTAATAAAATATGAATAAATTAAAACCTATTTGCAGACTTATTTATAAAAATATCACAGTAAATCCTATTCCAAATTATCAGAAAGTTAAACATTGTATGCTTTTTTCAAAAGATCAGTCTTTTAAGTGTTTCTTGAATAAATTAATAGTATCTAAACTTCTAAGATCTAATGGCAGATCATTCCAGAGTTTAGGTGCAGCAACGGAAAAGGCCCTGTCTTTTGACCTCTTATTTGACCTCTTACCACCCACACTTTAGCTTGTAAGCTGGCTCTCATATTTGGGTTTTGCCTTACAGCCCCTATCACACACACAATTGCCTATTATGTCATTGGCAGCCCCTTATGGAAAAAGTTATTGATACAGGATTCCTGTGGTCCTTGAAAGTCCTTGAATTTAATTTTTTGTTTTCAATGACTTGAAAACCCTTGAATTTAGACTCAGGTTCATCAAAATTTCTGAAAAGTCCTTGAAAATAAGTGCAGATGAATCAAAGTTTTCTTTTGTTGCCGCTGCTAAGAGTGTAAGTTTCTAGCACAAATTGCGGAAAAATGTTTTCCTTTCAAATCCCATATGGGATTATAATGTTTGATACATGATTTTTATATACCATATTTATTGATCTATAAGCCAAGTGATTTTTTCCCAAAATTTCACCAAAATCTCAAAAATTTGAGCTAGAGAAGGGTTCTTGGCTTATAGCCAAGTGTTTTTCCAAAACATAATTTTTTTCCAAACATAATTTTTTCAAAACAGCAAATTCAAAGGAAACATAAAGATATATCCGTCTATAAGCCGAGATATTGCATGCAGAATTAAATAACAAAACAAGTTGAAGTTGGTCATTGACTTTATTTATGTGGTTGCTTCCAAAGGAGCAGTTTGTTGTTTTCAGCGTGTAGTAAATACGGCTTACTGTTCATCCTCGGTGTCGGTGTGGAATTCATCATCCATCTCCTCCTTATGTTCTAGTTCTGCCATTTCTTCCGAGTAGATTGCTTAATCTTCCATGCCATCTAGAGTAATTAACAATTCTGTGGGTTTTGAACGAGTATATAGATCTTGCAAAAGTGTTTCAAGAATTTTGAAACTGACCAATCAGAATTGCCCATTCCCATGATTGGTAAACAATCTCGTCACATTTTACGGTGTTGAAAGTTGCAATCACATGAAAAGAAAACTTACCTTGGCTTCTGAGCTCTGTAAACCAGTAAAGTGATCTCTCATCCAGCCGCAGATATTTCGGAGTGTAACGGCCCATCATTGTGCGTTTCGCTGATATCTCGAGCTCTCCTTTTAAGAGATTCTCTTTTGATCTTTCCTCTAGCGACTCACTGATGACTTACTCTCTGGTTATCTCGGAATTGTTTTCGATGGCAATGCTGCATTGAAACTCGTTTCTGTTTTGAAACTTTTACATCCATTTCAGTTTGTTCAGCTTATAGCCGAGTATTTTCAATTTACATGTTTTAAGAACTTTCCTGATTAAAACAGACCGAAGTTTAGGGTCTCTGGTCATAGCCGAGATTGGCTTATAGATGAATAAATACCGTATGTTGTACCTGATTGGTGCGTGAAAATGACCTTGGTTACCTAGCGTGCGAACACTCTGGTGCGCATGCTTGAAGTCACGTTCGTTGAACTTTTTGGCCAGTCCTGCATGCAACTGTTTTTTATTTCTATTAGCTCCTATTTAATGCAAAAAGCCCTTAAATTTGAGAGCCAAGGTCCTTGGAAGTCCTTGAATTGGGTTCCTGTTAAACTGCAAGAACCTCGATGATACCTCTGATCCTCAAGTAAATAAATTAACAAGAGGTTTGAAAAATTCTTTCCATACAAAAGTAAAACCTAAACTGAAGTCAAAATTAGTTAACCCTTAATCTCAGACCAGTGATCAAGATAGAATTTCTCATTACAATATCAGTACAGTTTCAAGCAGGTAAGTGATGAGATATGTAGAAAAATATAAATCAGGGGATTATTGGTTGACCCAATACCAAATTCTCCAAACTCACATCATAAGAATTGTATAGAAGATAGTAAGAAGATCGTAGTAGTAGCAAGTAGGATAAATATTGTAAGAAATATACAAATTTTTCTAGAGACATGTTTCGGCATGCTTATGCCATCTTCAATCAATAAGTAACTGAACGTTGATTGAAGATGGCATAAGCATGCTGAAACATGTCTCTAAAAAAAGTTGTATATTTCTTACAATAGTAAGAAGAATTATGAATGAGATTTGGGGAGTGAAAGGGTTAAGTGTAAAGATTGTTTCATGTGCTTATTATTGAAATGTGTTTATTCTTTTGAGTGTTAGAACAATAATTTAATTTCTTTTTTTTACAAACTGGTTTTCAGGGTTACTGCACTCTTTGAAAGTCCTTGAATTTAAGTCTTTGTTTTCAAGGACTAGAAAACCCTTGAGTTTAGTCTCATGTTCTTGAAAATTTCCGAAAAGTCCTTGAAAATAAGTGCAGATGAATTGAAGTTTTCTTTTTTTGTTGGTGTTGAAAGTGTAAATTTCTAGCACAGATCACAAAAAGTAGTTTTTCTTTCAAATCCTGTGTCTAATTATGATGTTTGACATGTAACAATGAGCCATGTTCTTTTATTTCTGTGAGTTCCTATTTAATGCAAAAAGTGCATGAATTTGAGAGCCAAGGTCCTTGAAAATAACCTAAAAAGTCATTGAAAGTCTTTGAATTGGGTTCCTGAAAAGGTGCAAGAACCCTGGGGTTTAGTTTGTAGTCTTGTTTCTTTGGGTGTGAGTACTATTAGCTTACATTTTTTGCAGAAATCAATGATCTGGAAAAGATGAAGAAGAAACTTGATGCAATGGAATCTGCTCTTGTTGTTGGTAAGTTGCTGAATAATAGTTTGCTCTCTTTCTTTTCCTGTTTGTTTGGTTTGCCTAGTTTTGCTTTCCACCATAGACCACTTTCATAAATGGCTGCTGGATTAATATTCTTATCAATAAAGATTTGGAAATGCAAAGACTGGCAAAAACCAACGATAAAAATGGATGTTTCGGTGTCATCATGACGCCATTGTCAAGGAGAAATAAAAAATTACAAAGTTTGTGGCAAGGTGTAACACTAAATTAGCATAAGTAAGTATATTAATATTAATATTCTTTTGTTTAAATTTAAATTAGCCCTTATGGCCTCATTCTCCAGTACAAAATTCAAAAAAATACATTATCTCAAGTGAGGCCAGAAGGGCTAATTTAAATTTAGACAAAAGAATGTTCATCCAGCAGCCATTTATGAAAGTGGTCTATTGCCAAGCTACCACAAATGGAAAATTATTTTTGCATTTTATGATATGCTAAAAATGAAAGTTTGCAGAACTGTCACATTGTACATTTTAAAGGTACGGGTACCTTATTTTAATACGCAATCACTAAAGGTTATGGGTCCTAAATGGTTAACTTTGTAGTGTGTTTTCTTCTGTTTAGATATGTAAGTCTGTCTGCACTGCAGCATTTTACAACTTAATAAACAATCTTTAAATATGTAGAGAAAATGAAGTTTGCTAGTTCATGAGATTTTCCAGAAATGTGGAACCATCCTTTGCCACTGACATTGGTTTTGGAATGTTTAAAAGTTGATGCAACATTTTGAAAACCTTTGTTTACATGGAAAAACTCACAGGCAGAGTGGGTTAAACTTGATGCTCTTAAAATTAATAGGCTTTCCTGAAAAATTCTTTAAAATTTTTGGAATTTTTAACTTGTTAAATGAAAAACGTTGGGAAACTGTACATGTTTGTTAACAATGGTAGCACCAAACATTGTCATGTGACTTACTTTTGTAACCAATTTTGTAGGTTTCTTTGAGAAGGATGAGGACCTTAAAACTGAATTCATGAAGATAGCTGATGAAATGAGGGACGATTACTACTTTGCACACACAACAAGTGAAGAAATTTTGGCAGATTTGGGCCATAAAGAGTATGTGAAGCATTTTGATTTTGATTACTCTAGACTGGTCATAGGGTCATTAAAGCCACATCTGGCATTTTTTCCTGTAAACAGTCATCTATAACTGTGGAGGGACTGTTCCATGTTTTTTATTTACTAAATACGTGCTTTTTCCTTTCAGTCAAGTGGTTATTTTCCGTCCCGCACACCTTCATTCAAAGTTTGAACCAAACAAAGAAGTCTTTACAGGAATGGTCGATGCTTTTTATATCAAGAAGTTTCTCAAAGCTTCAGTCCATGGACTCGTCGGTCACTTGACTCCAGATAATGAAGATCAATTCAAGAAACCCCTGTGTACCGTTTATTACGATGTTGATTTTCAACTTAATGCAAAAGGTACTAACAGTTCATTCTATCTTGTCTAAAATGCAACCATCTGTGCTGAGCCAGGTGAAAAATGTAAAAAAATGAGACGATAAGATGTGTTCTGAGCAAAGAGGGATCCCGATCCTGTTTCATGCACAAAGTTAGGAAATGTTCAAACATCACTTTTACTTCAAAATCTTTTTTTTTTATGTACATCACAAATTAAAAGGGGAAAACTGCAAATCTCAGTGGGCCTGCCTTTTTGTAAAATTAGTGTATCACGGATCAATGTTGTGCATACTAGCACGTCAAGTATAAATCTTTTGCCACCGTTCTTATGCTTTGCTCAAAGGTTCAGTAGCTGCCAATTGAAAGTGTTTTTTAGTCTTATTTCTTACATTTCCCTACCTTGATTGTAAACCCTTGTCTCTAAAATTTGGCCAAAGAGCGTAGTATTATCTTCTCCATGGTGCAGATGTTGAAGTGACTTTTGATTGCCTGATACAGGTTCTCAGTTAGAGAAGATAGGCAAAAAAAAGTGGCTGAGCAAATGTTGGATAAATTAACAATGTGTAGCTTCAGTAGAATAACCAATTTCTTGCTCCAGAATGTGGGAAATGCAGATGATTTTTTAATTTTCATGTGGGAGCATGTCTCTGCCTCTTATCTCGCTTTTTCTACTCACTCTGTTTCATATTGAAAAAAAAACTTGTTTATTTGCCAATGCATAATGAATGGGAACAAGTGGTTGACATTTACAAGTAACTGCAAACATCAGATAGTCTTTAAAAGGTATATAAAGTTTTAGATCTGTAGGCTTGAAGAAATGCATTCTGTGTTAGAGTTCCCAGTTACACTTTTATATTCAACTTGTCGGCAATCTTACCATCTGCAGGTACACGATATTGGCGTAATCGAATTCTGAAAGTTGCTGCCCAGTTCAAGAACAAAGTGATGACCTATGCTTTTGCATCCAAGAGTGAATTCTCTGGAAAACTTGACAAATTTGGTTTATCCTCTGAGGGTAATGATGTTTTGGTAGCTATAGTGAGTGATGATGGAATGAAATATCCCATGAAAGAAAAATTCAGGTAGATATATACATTTGTGGGCTGTGGTGTGTTGTGCTCTACAGTGCTTTTTTTTTTCTTGTGCTGTGCTTTGTGTACAAACTCTGTTCTAACCAAGTCCTCCTTTTGACACAAGTTTGGTATTGACAAGAGTTGTTCATTAACCCTCTGCATTTTGCAACATATTACTTTTACTGCTGTACTCTACAACTTACTATACAACATGTGAAAGTGTAGTGTTATCGTTCAGGTGAGTGTACTCCTGAGAAGGACTGTTGTTGGTTGTAGTGACTGACGTTTCAACAACCTGTGCCAATAACATCAGGGCAAAACTGACCAATCATTTTACATGAACTGAAATAAGAACATTCTACTGACAACAACTATTACTACTTGACTCTGATGATGACTTCTGCACAGGTTGTCGAAACATCAGTCACTACAACCGACAACAGTCCTTCTCAGGACAACACTCGCCCGGACGATCACACTACACTTTCACATGTTACCCCTGGGTTCAAGCCATTTACTGCACAACTATACAACAGTAAGCACATGTAGTCAAGGATTTCCTTTTATTTAAGTTGACCTTCCTAATCCACTCCTCCTGGATATGGAGAATTTGGATGCTGATATTAGGTGGTAAAGGGTTTATGGTTGTTTGACTTCTTCTGCTGTTCTTCACAGCCTGACATCATGAATCGCTGTTTACTTTTCAGTGTGGACACCTTCAAAGATTTCCTCACCAACTACTTTGCGGGAAACATTGAACCTTACATCAAATCTGAGCCAGTACCAGAGACCAATGATGGCCCTGTGAAGGTAGGGTGTGGGTTATGTTAATGTAAATACCCTCATTAGACAATTGACTACTATTTATAGAGGTGAAGGTGGCAAGTGATGTAAATATCTGCTGCTAGCCCCTGACACAGAGGTGAAGAGTTGTTTTCATACATATCAAAAAACAGTGAGATAACATAGCAGAGAACGATGATTTTAACTCATTTATTCTTACAACGATTGCAATATTTTTGGGCTCAAACCCTGTGTGAGTTGTTCAGAGCTGAATAACAGAGGAGTTCGAGTGGCCAATCAGAGGGCACTTTCAATGCTATTCACTGTTTTAGTATTTACTGACTACCCCTGCTAGGACTGTTGCTTATATTTTTCTAAGTACAAGAACACTGAGTTGTCTATTAATATAATGTATTAAAAGTAGTGACTAAATTTTTGGTCATTCAATGGTTGACCAAGACAGAATTTCTTCTTACAATATCAATACAATCGCAAGCAAACAAGTGATGGGAATAAAGAAAAATGATAATTATAATAGTAATAATAATAATAATAATAATAATAACAATAACAATAATAATAATAATAATAATAATAATAATAATTGTTTTGTTTTTGTTTTTGGTTTTTGTTTTTTTGTTTTTTGTTCAGTATATAGATTTTGATTTCACTATAGTTAATGGGCTATGCTCAAGCGCCCCGTCATACTATTCTTATTGTATATAGCATACAAAAGTTTTAAACTGCTGCATAATAATTATTTTATTAATTTCTATTGCGCATTACAATATGATCTTAATACAAATATATAAAAATACAAATGTAAATGTAAATTTAAATATAAATACAAGTAGGAAATATAAAATATATATATGATAAAAAAATAAAATAAAATAAAAATAAAACAAGCATCACATCTAACACTATAAAAAAGCTAGTCTAAAAAGATACTAACAAATACTAAATTCTCCAAGCTAACGTCATAAGAACCGTATGGCAGACAGTAAGGAGAATTATGAATGTGATCTTGGGAGTGAAAGGGTCAGAATTTCATGGAATAAAACATTTACAGACGACTCTGTATTAAATGGGACCGTATTAAGCGGTCACCGTGCATGAAGCGGTCGGTTGTCAAAGTCCTGAAATTATAACCCCCTTGATTTTTTCTAAGCAACATATGGCAATCTGCGGTCTTTGCTTGCACTTAGTGGCACGGAAAGTCGTAAAAATCTTAAACAAAAGCTCATCTTTCAAACCGGCAGTCTAAATCCCACAGGGATCAACGAACGCTTTTCATTCCACTAATTTA
